# Age, sex, and cell type-resolved hypothalamic gene expression across the pubertal transition in mice

**DOI:** 10.1186/s13293-024-00661-9

**Published:** 2024-10-24

**Authors:** Dustin J. Sokolowski, Huayun Hou, Kyoko E. Yuki, Anna Roy, Cadia Chan, Wendy Choi, Mariela Faykoo-Martinez, Matt Hudson, Christina Corre, Liis Uusküla-Reimand, Anna Goldenberg, Mark R. Palmert, Michael D. Wilson

**Affiliations:** 1 Genetics and Genome Biology, SickKids Research Institute, Toronto, ON Canada; 2 Department of Molecular Genetics, University of Toronto, Toronto, ON Canada; 3 Developmental and Stem Cell Biology, The Hospital for Sick Children, Toronto, ON Canada; 4 Donnelly Centre for Cellular & Biomolecular Research, Toronto, ON Canada; 5 Department of Cell and Systems Biology, University of Toronto, Toronto, ON Canada; 6 Division of Endocrinology, The Hospital for Sick Children, Toronto, ON Canada; 7 Departments of Pediatrics and Physiology, University of Toronto, Toronto, ON Canada; 8 Department of Computer Science, University of Toronto, Toronto, ON Canada; 9 Vector Institute, Toronto, ON Canada; 10 CIFAR, Toronto, ON Canada; 11 Institute of Medical Science, University of Toronto, Toronto, ON Canada

## Abstract

**Background:**

The hypothalamus plays a central role in regulating puberty. However, our knowledge of the postnatal gene regulatory networks that control the pubertal transition in males and females is incomplete. Here, we investigate the age-, sex- and cell-type-specific gene regulation in the hypothalamus across the pubertal transition.

**Methods:**

We used RNA-seq to profile hypothalamic gene expression in male and female mice at five time points spanning the onset of puberty (postnatal days (PD) 12, 22, 27, 32, and 37). By combining this data with hypothalamic single nuclei RNA-seq data from pre- and postpubertal mice, we assigned gene expression changes to their most likely cell types of origin. In our colony, pubertal onset occurs earlier in male mice, allowing us to focus on genes whose expression is dynamic across ages and offset between sexes, and to explore the bases of sex effects.

**Results:**

Our age-by-sex pattern of expression enriched for biological pathways involved hormone production, neuronal activation, and glial maturation. Additionally, we inferred a robust expansion of oligodendrocytes precursor cells into mature oligodendrocytes spanning the prepubertal (PD12) to peri-pubertal (PD27) timepoints. Using spatial transcriptomic data from postpubertal mice, we observed the lateral hypothalamic area and zona incerta were the most oligodendrocyte-rich regions and that these cells expressed genes known to be involved in pubertal regulation.

**Conclusion:**

Together, by incorporating multiple biological timepoints and using sex as a variable, we identified gene and cell-type changes that may participate in orchestrating the pubertal transition and provided a resource for future studies of postnatal hypothalamic gene regulation.

**Supplementary Information:**

The online version contains supplementary material available at 10.1186/s13293-024-00661-9.

## Abstract (significance)

The hypothalamus is required to initiate puberty and develop secondary sex characteristics. While several hypothalamic-expressed genes are known to be essential for puberty, the gene regulatory networks that regulate its timing are not well established. Since puberty begins earlier in the male C57BL6/J mice compared to females, our study set out to identify puberty-related genes by focussing on genes whose pattern in expression was conditional on age and sex. We first used RNA sequencing to examine sex-biased developmental trajectories in the mouse hypothalamus, where we tracked gene activity in the hypothalamus at two prepubertal, two peri-pubertal, and one postpubertal timepoint. To address an inherent limitation of using bulk RNA-seq profiling of the hypothalamus, we integrated single-cell RNA sequencing and spatial transcriptomics data to identify which cell types most likely give rise to the observed gene expression patterns. Some of the genes we found are part of specific pathways related to hormone production, nerve cell activity, and the maturation of support cells in the brain (glial cells). By combining bulk RNA-seq data with single cell RNA-seq we inferred an increase in the maturation of cells that form the myelin sheath (oligodendrocytes) coinciding with pubertal onset. In summary, this study highlights gene expression and cellular composition changes that occur in the hypothalamus during postnatal development in a manner conditional on sex. This work should serve as a resource for hypothalamic gene regulation during postnatal development in mice.

**Supplementary Information:**

The online version contains supplementary material available at 10.1186/s13293-024-00661-9.

## Highlights

Gene expression in the hypothalamus in male and female mice was obtained at five postnatal ages spanning pubertal development.

A set of genes influenced by sex and age were identified and found to be related to pubertal development.

Combining these results with single cell RNA-seq and spatial transcriptomic data we profiled puberty-relevant neuronal and glial gene expression signatures.

A hypothalamic gene expression resource for male and female mouse was generated to facilitate access to this data.

**Supplementary Information:**

The online version contains supplementary material available at 10.1186/s13293-024-00661-9.

## Introduction

Puberty is a fundamental period of mammalian development when individuals reach sexual maturity. Despite being a nearly universal event, pubertal timing within the population varies and is known to be influenced by genetic and environmental factors [1–4], though much of its variation remains unexplained. Rare mutations in several genes lead to pubertal disorders such as central precocious puberty (CPP), defined as abnormally early pubertal initiation, and hypogonadotropic hypogonadism (HH), defined as delayed or absent puberty [5–7]. Genome-wide association studies (GWAS) investigating the age of menarche in females and age of voice breaking in males [8] have identified shared and sex-specific sequence variants related to pubertal timing in the general population. Furthermore, early puberty is associated with an increased risk of later life health outcomes such as cancer, diabetes, and cardiovascular disease, while late puberty is associated with an increased risk of osteoporosis and mental health disorders [9]. Importantly, environmental factors such as diet, body mass index (BMI), prenatal growth, and psychosocial experience are associated with differences in pubertal timing [9]. Due to the genetic and environmental impact on pubertal timing and development, animal models where genetic and environmental background can be controlled, are of great use for understanding this process.

Puberty is initiated in the hypothalamus by pulses of gonadotropin-releasing hormone (GnRH) increasing in frequency and amplitude, which then stimulate the pituitary gland to increase secretion of luteinizing hormone (LH) and follicle-stimulating hormone (FSH). This cascade begins an organism-wide feedback loop involving many genes, cell types, and gene regulatory mechanisms [10]. Previous studies have investigated hypothalamic regulation during puberty and have discovered a growing list of gene-regulatory mechanisms that can directly regulate pubertal timing [11]. These studies include the epigenetic mechanisms activating and inhibiting pubertal onset and spatial transcriptomic programs associated with postnatal development in the female rat hypothalamus [12, 13].

Puberty is an inherently sex-biased process that results in the development of secondary sex characteristics in males and females. Additionally, males and females undergo pubertal timing at different ages. In humans, puberty typically occurs earlier in females, and in some rodents (such as the inbred C57BL/6J mouse strain used in this study), male mice undergo puberty earlier than female mice [14]. Despite the sex-specific physiological differences related to puberty in males and females, GWAS studies looking for genetic factors related to the timing of pubertal onset (age at menarche in females and voice breaking in males) revealed that the significant genetic variation associated with pubertal timing is mostly shared by males and females [15, 16]. For example, in humans, the same variant in the *LIN28B* gene is associated with puberty-relevant phenotypes in both males and females [16]. Nonetheless, understanding the biological consequences that specific gene expression programs have on puberty is complex. For example, *Lin28b* and *Lin28a* knockout mice revealed sexually dimorphic phenotypes related to body weight and pubertal development [17]. For this reason, studying the hypothalamus in males and females of different species, at various developmental stages, and under various environmental conditions has been essential for understanding how pubertal timing is controlled [17].

Identifying genes whose developmental trajectories are offset between male and female mice should be enriched for candidate genes that influence or are influenced by pubertal onset. However, few studies have characterized hypothalamic gene expression across the pubertal transition in both males and females [17]. We previously utilized microfluidic qPCR to measure the expression of the mouse orthologs of 178 puberty-related disease genes (e.g. *Mkrn3*, *Dlk1*) and candidate genes puberty-related GWAS associated genes in the hypothalamus, pituitary, gonads, pineal gland and liver at prepubertal (PD12 and PD22), peripubertal (PD27 and PD32), and postpubertal (PD37) times [18]. We found that most temporal gene expression changes in the hypothalamus occurred before puberty and that relative to the pituitary gland, few sex biased genes were detected [8, 9]. Mirroring the pubertal onset differences between males and females, the prerequisite puberty genes *Gnrh1*, *Kiss1* and *Tac2* increased in expression between PD12-22, whereas the same genes in females increased later PD22-32 [8]. Together, genome wide profiling of hypothalamus gene expression could reveal additional genes following similar patterns.

Single cell genomics technologies now allow for the ascertainment of gene expression profiles from individual cell types within the hypothalamus [2, 19, 8]. For example, a scRNA-seq study focusing on the prenatal development of the mouse hypothalamus samples several timepoints, including two postnatal times points (PD14 and PD45) [3]. While incredibly valuable, cost restraints and technical limitations still limit the number of samples profiled and genes detected in a single study. For this reason, computational approaches to integrate scRNA-seq data with RNA-seq collected from bulk tissue can allow one to harness the advantages of both types of data [5, 6].

In this study, we measured hypothalamic gene expression in male and female mice at five timepoints spanning pubertal transition. We identified genes whose expression is conditional on both age and sex and found several established puberty-related genes as well as additional genes whose role in pubertal control requires further investigation. Using a hypothalamic snRNA-seq with two analogous timepoints to our study [3], we mapped the age and sex conditional genes to their most likely cell type of origin. From our bulk RNA-seq data, published snRNA-seq data and spatial transcriptomics data, we inferred that substantial oligodendrocyte expansion occurs prior to pubertal onset. To enable the use of this gene expression dataset in further studies, we created an interactive Shiny App (wilsonlab-sickkids-uoft.shinyapps.io/hypothalamus_gene_shiny/) as well as downloadable resources. Overall, this integrative analysis of the hypothalamic transcriptome incorporating age and sex serves as a resource for understanding hypothalamic gene regulation during the pubertal transition.

## Materials and methods

**Animal and tissue collection.** We collected the hypothami of 48 C57BL/6J mice at PD12, 22, 27, 32, and 37 in males and females (4–5 mice per age/sex). Tissue dissection and RNA extraction follow the protocol in Hou et al., 2017 as this study made use of the same isolated RNA [8].

### Library preparation and sequencing

RNA-seq libraries were prepared using an automated QuantSeq 3’mRNA-seq (Lexogen GmbH, Vienna) and Agilent NGS Workstation (Agilent Technologies, Santa Clara) at The Centre for Applied Genomics (TCAG) (Toronto, Canada) as per the manufacturer’s protocol (UTRSeq). The automated QuantSeq 3’mRNA-seq library construction was described in detail in Hou et al., 2022 [9]. Briefly, 250 ng of total RNA spiked-in with ERCC Spike-In Control Mix 1 (Ambion) as per the manufacturer’s protocol was used to generate cDNA. cDNA was amplified with 17 PCR cycles as determined by qPCR analysis using the PCR Add-on kit (Lexogen). The resulting libraries were quantified with Qubit DNA High Sensitivity assay (ThermoFisher). Fragment sizes were analyzed on the Agilent Bioanalyzer using the High Sensitivity DNA assay prior to sequencing. Single-read 50-bp sequencing was performed at TCAG on an Illumina HiSeq2500 Rapid Run or V4 flowcell (Illumina, San Diego) with cycles extended to 68 bp.

### Read processing

Reads from technical replicates were merged prior to downstream analyses. Fastqc (http://www.bioinformatics.babraham.ac.uk/projects/fastqc/*)* was used to examine the quality of sequenced reads. A customized script to trim both the polyAs and adapters at the end of the reads [9, 10] was used. The script implemented a “back search” strategy to account for cases where a mixture of adapters and polyAs were seen at the end of the reads. In addition, the first 12 nucleotides were trimmed with Cutadapt [9, 10] based on the manufacturer’s recommendations. Only reads longer than 36 bp after trimming were used for future analyses. After trimming, Fastqc was performed again to examine read quality, and over-represented reads, namely reads mapping to *BC1* (brain cytoplasmic 1), were removed. Trimmed and filtered reads were aligned to the genome using a splice-aware aligner, STAR (version 2.5.1b), with default settings except “--outFilterMismatchNoverLmax 0.05” for QuantSeq [11]. Quality control (QC) of mapped RNA-seq reads was performed using Qualimap version 2.2.1 [21] (Supplementary Table S1). Read signal was visualized with the UCSC genome browser [12]. Reads were assigned to genes using featureCounts (version 1.5.3) [14] with parameters “ -s 1 -Q 255 -t exon -O”. Gene models were obtained from GENCODE M11 [15, 16].

### Count processing and evaluation

Counts successfully aligned to GENCODE M11 [15, 16] were normalized based on ERCC spike-ins using RUVseq [17]. Genes with fewer than 5 reads were removed before upper-quartile normalization was completed with the betweenLaneNormalization() function [17]. Finally, ERCC spike-ins were used to normalize counts using the RUVg(), yielding the final normalized count matrix [17]. All samples were correlated to one another using Pearson’s correlation of all genes before being plotted with the ComplexHeatmap package [18]. Genes overlapping the RNA-seq and qPCR data of the same samples [8] were correlated using Pearson’s correlation analysis. Principal component analysis (PCA) of samples was performed with the “prcomp()” function [22] before being plotted with the ggplot2 package [23].

### Differential expression analysis

Pairwise differential gene expression analysis was completed across ages and sexes. Differentially expressed genes were calculated using the DESeq2 R package [24]. Genes were considered differentially expressed if they had a false discovery rate (FDR)-adjusted p-value < 0.05 and an absolute-value fold-change 1.5. Sex comparisons were completed at each timepoint, while age comparisons within each sex were completed between days 12 and 22, 22 and 27, 27 and 32, and 32 and 37.

### Varimax rotation principal component analysis

Principal component analysis (PCA) is a dimensionality reduction technique used to reduce every individual mouse’s global gene expression pattern into a smaller set of orthogonal vectors [22] (N_components_ = Nmice = 48). Varimax rotation decreases the distance between PCs and mice by adjusting the PC axes such that samples will more closely align with one varimax rotated PC (vrPC) [25]. By leveraging vrPC scores, defined by the location of a sample of a PC axis, we identified which vrPCs are associated with age, sex, and an age-by-sex interaction by completing a two-way ANOVA of timepoint and sex on vrPC scores. By leveraging vrPC loadings, defined by the association between a gene and PC, we measured which genes are represented by individual vrPCs.

Normalized count data and PC scores were used to generate varimax-rotated PCs with the “varimax” function in R [25, 26]. Varimax-rotated PC loadings and scores were acquired using the pracma package [25, 26]. A loading is a gene’s coefficient to the vrPC, while the score is a sample’s coefficient to a rotated vrPC [25, 26]. The association between scores, age, and sex was measured using two-way ANOVA. Multiple-test correction using the FDR was applied using the p.adjust() function in R [27]. The FDRs of the vrPCs with an associated main effect or interaction were plotted with ggplot2 [23]. We designated that genes with loading greater than three standard deviations from the mean loading are associated with a vrPC. We picked three standard deviations by inspecting a qqplot of loadings with the qqnorm() function. Genes associated with a vrPC were re-ordered by the loading magnitude for downstream analysis.

### Pathway and human RNA-seq enrichment analysis

Pathway enrichment of fused gene lists (e.g., PD12 vs. PD22, males and females) was completed using the ActivePathways R package [28]. Briefly, ActivePathways takes the p-values from different related gene lists (e.g., PD12 vs. PD22 - males, PD12 vs. PD22 - females) and fuses them using Brown’s extension of Fisher’s method (i.e., Fisher’s combined probability test) [28]. Then, it computes pathway enrichment of each individual gene list and the fused gene list using a p-value-ranked Hypergeometric test [28]. The resulting statistics provide pathway enrichments annotated to each DEG list and their integrated p-values [28]. In our study, ActivePathways yields four levels of enriched pathways: male-only, female-only, male and female – independent (i.e., enriched in males and females without fusing p-values), and male and female – dependent (i.e., enriched in both males and females after fusing p-values). We used the “Mouse_GO_AllPathways_no_GO_iea_September_01_2022_symbol.gmt” gene set database from (http://download.baderlab.org/EM_Genesets/*)*, which systematically curates a gene set list from multiple sources (Gene Ontology, Reactome, Panther, etc.) as our pathway enrichment database [29].

Pathway enrichment for gene lists without p-values following a multivariate normal distribution (i.e., vrPC-associated genes, oligodendrocyte-pseudotime associated genes) was completed using the gProfileR R package using an FDR correction, with genes detected in the RNA-seq dataset as the custom background and with GO: BP, GO: MF, and GO: CC being queried [30]. Here, “genes” represent oligodendrocyte-pseudotime associated genes or associated loadings ordered by FDR-adjusted p-value or vrPC loading, respectively. Biological pathways identified by integrating developmental changes across sexes were completed with ActivePathways [28].

We used the Differential Expression Enrichment Tool (DEET) to compare our age-biased DEGs and vrPC-associated genes to 3162 consistently reprocessed sets of DEGs derived from The Cancer Genome Atlas (TCGA), Genotype-Tissue Expression Consortium (GTEx), and from various studies within the Sequencing Read Archive (SRA) [31–35]. We ran the DEET_enrich() function to measure which of our age-biased DEG lists and vrPC-associated genes enriched for publicly available human DEG sets. DEET_enrich() also identifies DEG comparisons whose overlapping DEGS also has a correlated fold-change, suggesting that the shared DEGs and pathways may be under shared regulation [36]. Correlation plots were generated using the DEET_enrichment_plot() with default parameters. Lastly, we enriched our neuron-neuroendocrine mapping age-by-sex associated genes with LepRb + cells in the hypothalamus by overlapping Trap-seq + genes from Alison et al. [37]. and testing for over-representation with a Fisher’s exact test. Pathway enrichment plots for ActivePathways, traditional pathway enrichment, and DEET, were completed using the DEET_enrichment_plot() and process_and_plot_DEET_enrich() functions in DEET [31].

### Processing of public hypothalamic scRNA-seq data

Filtered gene-barcode matrix files for the PD14 and two PD45 samples were downloaded from the Gene Expression Omnibus (GEO) series GSE132355 (P14: GSM3860745, P45-rep1: GSM3860746, P45-rep2: GSM3860747) [3]. Counts were processed and integrated using the process_dgTmatrix_lists() function in scMappR, including all genes and scTransform as options [5, 38–40]. Briefly, process_dgTmatrix_lists() is a wrapper for Seurat V4 and scTransform [5, 38–40] before cell-type labeling with cell-type enrichment of the CellMarker and Panglao databases [41, 42]. In our preprocessing of these data, we used the Integration Anchors with Canonical Correlation Analysis, a rigorous recommended batch correction method [43]. because the PD14 mice were from the CD1 strain and the PD45 mice (male and female samples) were from the C57BL/6J mice (male only samples) [3]. While we may have lost some developmental signal through this rigorous batch correction [44], the major cell-type markers and developmental trajectories we observed would be more reliable and translatable to our bulk RNA-seq.

Cells were first labeled with the cell types provided by the original authors [3]. we further applied the cluster labels and cell-type markers generated from process_dgTmatrix_lists() [5]. to provide further specificity to these cell-types. For example, “oligodendrocytes” contained clusters “4”, “24”, “17” and “24” which could be annotated to “oligodendrocyte precursors”, “developing oligodendrocytes”, and “mature oligodendrocytes” [45]. Cells with a different major cell-type label (i.e., neuron vs. glia) between the original author and this analysis and cell-types whose markers were primarily mitochondrial genes were discarded for differential, proportion, and trajectory analyses.

### Age-biased cell type-specific gene expression and cell-type proportion in scRNA-seq data

Age-biased cell-type proportion changes were measured with Fisher’s exact-test [46]. Age-biased genes within each cell type were measured using the Model-Based Analysis of Single-cell Transcriptomics (MAST) using the FindMarkers() function in Seurat [38, 47]. We filtered genes with an FDR-adjusted p-value < 0.05 and required the gene to be expressed in > 25% of the cells in either age group.

### Cell-type deconvolution

All defined cell types and all samples were used in cell-type deconvolution analysis. We completed RNA-seq deconvolution with DeconRNA-seq [48], Digital Cell Quantification (DCQ) [49], Whole Gene Correlation Network Analysis (WGCNA) [36], Cibersort and CibersortX [50, 51], Cell population mapping (CPM) [52], MuSiC R package [7], and BayesPrism [6]. For all methods, the bulk RNA-seq dataset were the same RUV-seq normalized counts [17] and the scRNA-seq data were the SCTransform-normalized counts [40]. Cell-type proportions from the MuSiC and MuSiC-NNLS methods were computed simultaneously with the music_prop() function, using default parameters [7]. We then correlated the predicted cell-type proportion at PND12 with the cell-type proportions of scRNA-seq data at PND14 and the predicted cell-type proportion at PND37 to the cell-type proportions of scRNA-seq data at PND45. We used MuSiC-NNLS, the tool with the highest correlation to the scRNA-seq data, for downstream analysis. For all downstream analyses, we estimated cell-type proportions with scRNA-seq PD14 and PD45 timepoints combined. We used DeconRNA-seq to calculate cwFold-changes in scMappR because it had the strongest correlation between predicted cell-type proportions and scRNA-seq cell-type proportions of the three allowed RNA-seq deconvolution methods for the scMappR tool, namely DeconRNA-seq, WGCNA, and DCQ [5]. We then used the cell-type proportions estimated by MuSiC-NNLS to assign genes to cell types because the cell-type proportion filter of gene–cell-type assignment can use any deconvolution method [5]. The association between cell-type proportion, sex, and age was measured with two-way ANOVA.

### Cell-type specificity of bulk differentially expressed genes

We used scMappR [5] to generate a signature matrix from the scRNA-seq data in Kim et al., 2020 [3] by using the generes_to_heatmap() function in conjunction with our previously labeled cell types. We then calculated cell-weighted Fold-Changes (cwFCs) for genes associated with varimax-rotated PC 16 with the scMappR_and_pathway_analysis() function before sorting each DEG into the cell type driving it with the cwFoldChange_evaluate() function [5].

We next used scMappR [5] to assign genes to their cell type of origin based on the differential expression of the genes between the conditions of interest in a specific cell-type. To calculate the cell-weighted fold-change statistic, we inputted the bulk fold-change from PD12 and PD32, as these timepoints have the largest distance in the varimax-rotated PC 16.

### Cell-type enrichment of neuronal subtypes

We integrated BayesPrism [6], a statistical technique to reweigh the gene expression signature to account for cell-type specific gene expression and cell-type abundance of each major cell-type of interest (e.g. neurons) with scMappR [5], a statistical approach to assign DEGs to their cell-type of origin, to identify neuron-specific genes with an age-by-sex pattern in gene expression before assigning them to their neuronal subtype of origin. First, we generated a matrix of estimated neuron-specific gene expression in our bulk RNA-seq data using BayesPrism. Briefly, we used our RUVseq-normalized RNA-seq data [17], the raw counts from our processed Seurat [38] object, and our labelled cell-types (neuron and neuroendocrine merged) to run BayesPrism [6]. For data processing, we followed the author’s tutorial (https://bayesprism.org/pages/tutorial_deconvolution) using parameters specified in the tutorial while selecting the “mouse” as our species of interest. Because we were not comparing tumour to non-tumour fractions, we changed the “key” variable from “tumor” to NULL. We then extracted the neuron-specific gene expression matrix from the output of the get.exp() function before treating it as a typical normalized gene expression matrix. As such, we repeated the varimax rotation analysis and identification of age-by-sex association vrPCs and genes through vrPC score and vrPC loading analysis using the same parameters and cutoffs as used in our bulk tissue. We performed this analysis twice: once with all timepoints and once with only the peri-pubertal timepoints (PD22, PD27, and PD32).

With neuron-specific age-by-sex associated genes identified, we used scMappR [5] to sort these genes into neuronal subtypes. Firstly, we clustered hypothalamic neurons and neuroendocrine cells in the mouse hypothalamus using the FindNeighbors() (dims = 1:10) and FindClusters() (resolution = 0.5) functions in Seurat [38], where we identified 11 neuronal or neuroendocrine clusters. We then calculated cell-type markers for each neuronal or neuroendocrine cluster against all other hypothalamus cell-types (i.e., other neurons and non-neuronal cells) using a Wilcoxon’s Test within the FindMarkers() function in Seurat [38]. We then labelled each cluster by cross-referencing their top markers to the CellMarker database and the “Protein expression and localization” and “Tissue RNA expression” subsections of the Human Protein Atlas [41, 42, 53]. After cell-types were labelled, we recomputed cell-type markers between neurons alone (i.e., excluding non-neuronal cells from the analysis) to identify the differences between neuronal subclusters. The differences in neuronal marker expression were converted into a signature matrix using the seurat_to_generes() and generes_to_heatmap() functions in scMappR [5]. We then computed the fold-changes between genes between PD12 vs. PD32 in males [24]. Lastly, neuronal age-by-sex associated genes were assigned their neuronal cluster of origin using the scMappR_and_pathway_analysis() and cwFoldChange_evaluate() functions in scMappR [5] with the same parameters as in the bulk RNA-seq count matrix.

### Cell-type trajectories of scRNA-seq data

Cell-type trajectories were measured in oligodendrocytes (oligodendrocyte precursors [OPCs], developing oligodendrocytes [DOs], and mature oligodendrocytes [MOs]) using the slingshot R package [53] with default parameters other than setting the “extension” parameter to “n”. The starting cell type in each trajectory was set as the most “PD14-biased” cluster, namely the “Oligodendrocyte precursor”. We analyzed which genes had expression patterns associated with pseudotime trajectories using the tradeSeq [54] R package. We used the minimum number of allowable knots from the “evaluateK” function to fit the negative-binomial generalized additive model with the fitGAM() function [54]. Then, we tested the association between genes and trajectories with the associationTest() function [54], and corrected p-values with the “fdr” correction. Genes with an FDR-adjusted p-value < 0.05 and a fold-change > 1.5 remained for downstream analysis. We used the predictSmooth() [54] function paired with the scale function to generate columns for heatmaps. We identified genes based on their overlap with mouse TFs from ENCODE [55], puberty GWAS [56, 57] genes, and genes associated with varimax-rotated PC 16. We plotted the expression of these genes along pseudotime with the Pheatmap R package [58].

### Spatial mapping of oligodendrocytes in the adult rodent hypothalamus

We investigated the spatial distribution of oligodendrocytes in the adult hypothalamus using the Allen Brain Cell Atlas (ABCA), which contains 3.5 million spatially resolved cells in the adult C57BL6/J brain [59]. We counted the number of cells labelled as “mature oligodendrocytes” in each subregion of the hypothalamus designated in the MERFISH-C57BL6J-638,850 dataset of the ABCA (knowledge.brain-map.org) as well as the number of total cells in each of these regions [59]. We then investigated the enrichment of mature oligodendrocytes (MOs) in each subregion of the hypothalamus by comparing the proportion of MOs in the subregion of question to the proportion of MOs in the rest of the hypothalamus with a Fisher’s exact test. We then applied an FDR correction to the p-values of the Fisher’s exact tests for each subregion in the hypothalamus. We computed a fold-change from the odds ratios of Fisher’s exact test (enrichment cut-off: fold-change > 2, FDR-adjusted p-value < 0.05).

### Spatial gene expression of the adult female rat hypothalamus

To investigate spatially resolved oligodendrocyte proportions in the postnatal female rat preoptic area, we used data from Zhou et al., 2022, which contains 10X-Visium spatial transcriptomics data [60] for the female rat preoptic area at a prepubertal (PD25), peripubertal (PD35), and post-pubertal (PD45) timepoints [4]. Specifically, we acquired aligned feature barcode matrices “filtered_feature_bc_matrix.h5” and annotation of cluster markers of the 10X-Visium data from the original authors of Zhou et al., 2022. We re-processed each sample individually using Seurat’s spatial vignette (https://satijalab.org/seurat/articles/spatial_vignette) with the parameters described in the vignette [61]. Once re-processed, we merged the three samples using Seurat’s merge and combined variable features from each sample into a new feature set, and then recomputed principal components with the combined feature set, neighbours, spatial clusters, and the UMAP using Seurat with the top 30 principal components [61]. Then, we matched our clusters to the clusters of the original study using a combination of overlapping markers and relative positions on each spatial plot [3]. We then used Seurat’s “Integration with single-cell data” pipeline from the same vignette to assign a probabilistic score of each major cell type in the hypothalamus to each spot in these, using the PD45 scRNA-seq data as the reference dataset for integration [3, 62]. We then used Seurat’s AverageExpression() function to compute the average predicted score across all spots in each region and sample [3, 62]. We normalized them by the maximum score across samples. This analysis yielded a cell-type-by-spatial region matrix of each sample populated by the normalized cell-probability score. We then compared the average cell-probability scores between timepoints to identify regions of the preoptic area with a higher ratio of oligodendrocytes in the PD45 rat.

## Results

### Global transcriptomic view of the postnatal mouse hypothalamus across the pubertal transition in males and females

**Fig. 1:**
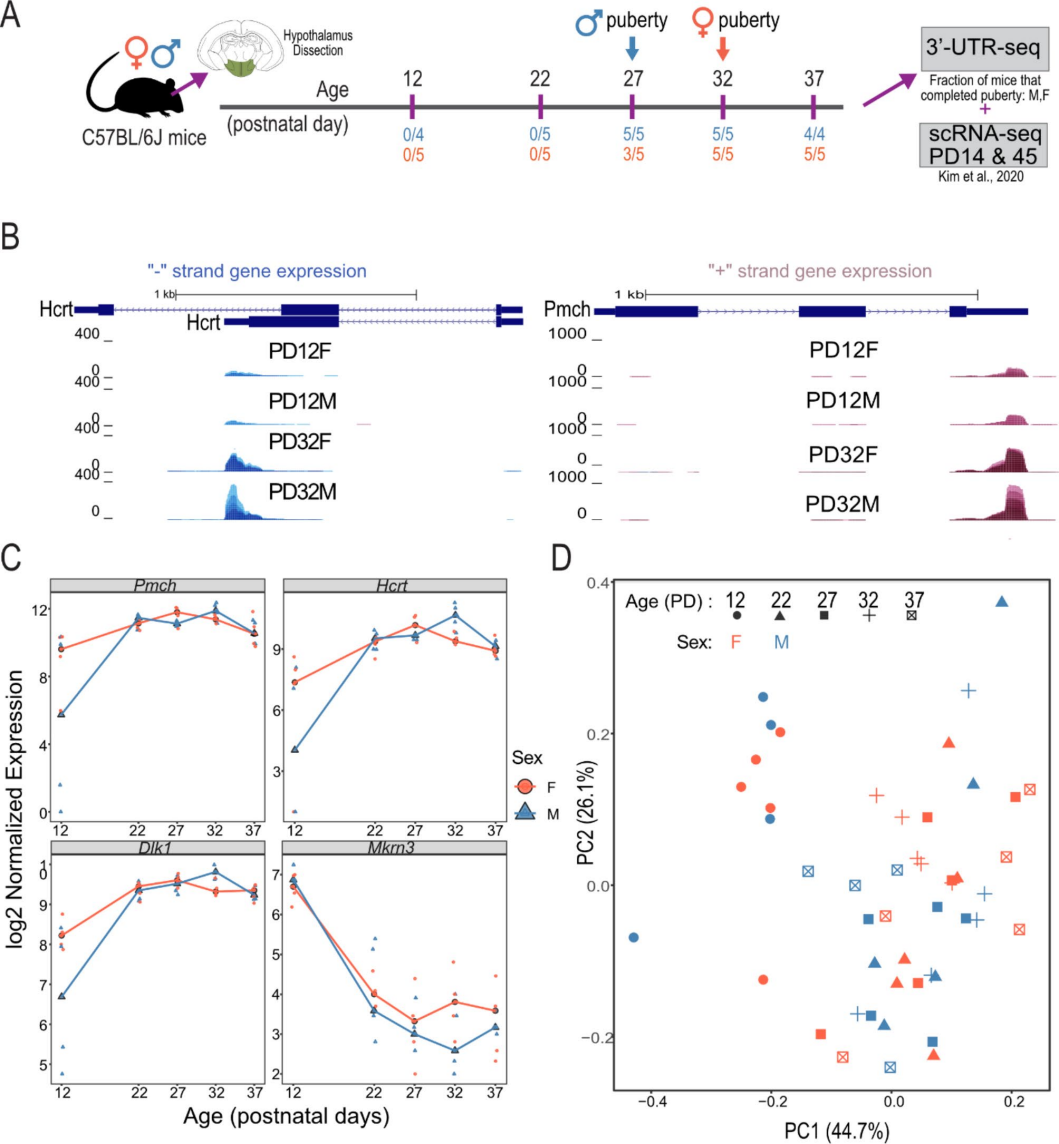
Overview of the hypothalamic mouse transcriptome at five timepoints across pubertal development in males and females. **A**) Schematic of samples taken across pubertal development. Whole mice hypothalami were dissected at postnatal days (PD) 12, 22, 27, 32, and 37 in both male and female C57BL/6J mice. Arrows dictate the average age of puberty in males and females, respectively. Extracted hypothalamus samples underwent 3’UTR RNA-seq. **B**) Genome browser of the *Hcrt* (top) and *Pmch* (bottom) 3’UTR at PD12 and PD22 in males and females. **C**) Distribution of normalized counts of *Pmch*,* Hcrt*,* Dlk1*, and *Mkr3* at every age and timepoint. The X-axis is age, and the Y-axis is log2-transformed RUVseq and ERCC-spike in normalized counts. Red lines and circles represent female samples, while blue lines and triangles represent male samples. **D**) Principal component analysis (PCA) of normalized gene expression across all samples and ages. The first two PCs are plotted with sexes designated with colour and ages designated by shape

To track the transcriptomic dynamics of the postnatal mouse hypothalamus, we measured genome-wide gene expression using 3’UTR profiling in male and female C57BL/6J mouse hypothalamus samples collected at five postnatal days (PDs) corresponding to early development (PD12), prepubertal (PD22), peri-pubertal (PD27 in males, and PD32 in females), and postpubertal (PD37) stages (*N* = 4–5 per sex/age) (Fig. 1A). We used an automated RNA-seq library preparation platform to generate 3’UTR libraries and sequence of all samples in a single batch.

To assess the quality of the 3’UTR-seq data we compared our data to microfluidic qPCR data previously performed for 183 genes using the same RNA [8]. Samples were highly correlated between the RNA-seq and qPCR data based on these 183 overlapping genes (R^2^ mean = 0.698, sd = 0.0270) (Supplementary Figure S1A). For example, four genes whose gene expression and expression patterns are well characterized in the hypothalamus (*Mkrn3*,* Cartpt*,* Dlk1*, and *Pomc*) recapitulated previously reported expression dynamics in the hypothalamus [60–64] (Fig. 1B, C). As previously shown with qPCR of selected puberty-related genes, PCA of the RNA-seq data revealed the greatest overall change in gene expression between PD12 and all other timepoints in both male and female hypothalamus samples (Fig. 1C). Furthermore, each sample in our 3’UTR-seq data is highly correlated to one another, with their R^2^ ranging from 0.89 to 0.99 between samples (0.98–0.99 between replicates) supporting that this dataset was of sufficient quality for downstream analyses (Supplementary Figure S1B).

### Pairwise differential gene expression across pubertal development reflects hypothalamic cellular composition dynamics and puberty-relevant transcriptional control

To investigate the transcriptomic dynamics in the hypothalamus throughout pubertal development, we identified DEGs between the studied age groups in male and female mice separately, as well as DEGs between sexes at each timepoint (FDR adjusted p-value < 0.05 and absolute fold-change > 1.5). We denoted age-biased DEGs with a positive fold-change to have higher expression in the later timepoint in development (i.e., PD12 vs. PD22, PD22 is greater). When comparing sexes, we designated DEGs with a positive fold-change to have higher expression in females than males.

**Fig. 2:**
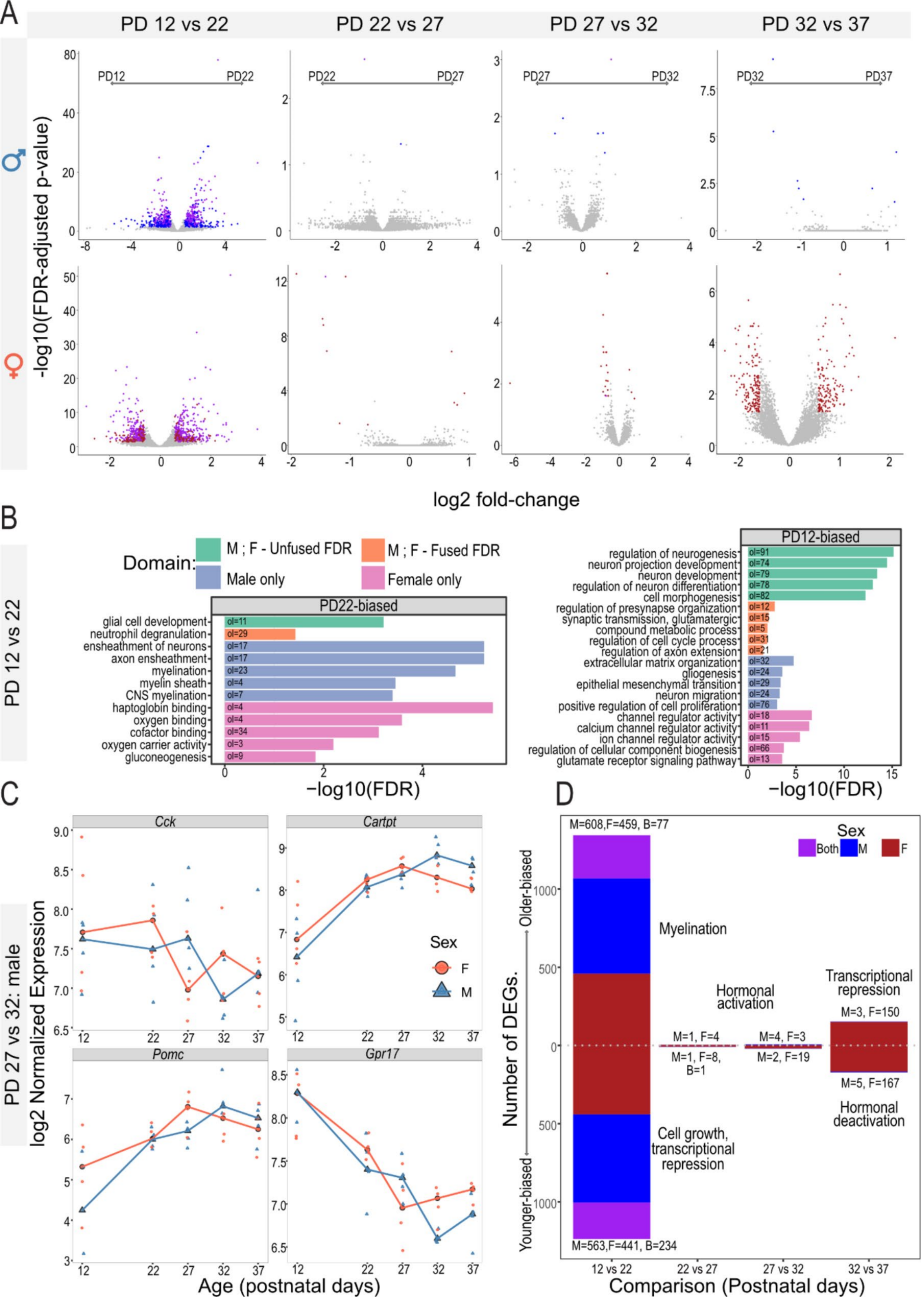
Differentially expressed genes (DEGs) across postnatal development in the mouse hypothalamus. **A**) Volcano plot of differentially expressed genes in each pairwise timepoint. The X-axis is the log2(fold-change) of the DEG, and the Y-axis is the -log10 (FDR-adjusted P-value) of the DEG as identified by DESeq2. Genes in grey are not detected as DE (FDR-adjusted P-value < 0.05, absolute fold-change > 1.5). Genes in blue are DE in males, genes in red are DE in females, and genes in purple are DE at the same timepoint in both males and females. **B**) Barplot of enriched pathways derived from DEGs between PD12 and PD22 in male and female mice. Genes are separated by upregulated and downregulated DEGs. Barplots show the -log10(FDR-adjusted P-value) of enrichment. Green bars represent pathways detected in both sexes, orange bars represent pathways detected by integrating sexes, blue bars represent male-driven pathways, and pink bars represent female-driven pathways. **C**) Expression profile of the four remaining DEGs. D) Barplot summarizing the number and major theme of pairwise DEGs across each timepoint. The X-axis is each timepoint, and the Y-axis is the number of DEGs. Positive genes were older-biased, and negative genes were younger-biased. **D**) Expression profile of DEGs involved in hypogonadotropic hypogonadism. The X-axis is age, and the Y-axis is log2-transformed RUVseq and ERCC-spike in normalized counts. Red lines and circles represent female samples, while blue lines and triangles represent male samples. Expression profile of differentially expressed GWAS genes (right). Row-normalized heatmap of GWAS-associated genes that were also detected as differentially expressed in our RNA-seq data Rows are genes, and columns are samples

We found that most DEGs are established between PD12 and PD22, before the physical signs of pubertal onset (vaginal opening in females and preputial separation in males). Approximately 32% (511/1560) of DEGs were shared by males and females (Fig. 2A). We looked for pathways enriched by these DEGs using ActivePathways [28]. Upregulated DEGs (higher expression in PD22 than PD12) enriched for pathways related to glial-cell development (Fig. 2B). Downregulated genes (higher expression in PD12 than PD2) enriched in pathways involved in cell differentiation, cell morphogenesis, and proliferation (Fig. 2B). These PD12-biased enrichments likely reflect that the brain (including the hypothalamus) rapidly increases in volume from birth until PD20. Accordingly, genes involved in growth would be more highly expressed at PD12.

We next overlapped our DEGs at all timepoints to a pre-curated list of rare-disease genes whose mutations lead to hypogonadotropic hypogonadism (HH) [60]. We found three overlapping genes, all of which were differentially expressed between PD12-PD22 and not at other timepoints. Specifically, *Il17rd* is downregulated in males and females (PD12 vs. PD22), *Sema3e* is downregulated in females, but not males, and *Rab3gap1* is upregulated in males and females (PD12 vs. PD22) (Supplementary Figure S2). Briefly, *Il17rd* is a member of the interleukin-17 receptor protein family and is involved in regulating growth through fibroblast growth factor and MAPK/ERK signaling [61]. *Sema3e* is a semaphorin, which acts as axon guidance ligands and organogenesis [62]. *Rab3gap1* is a member of the Rab3 protein family, which is involved in endoplasmic reticulum structure and has also been implicated in the proper development and migration of neurons [63].

We detected 317 DEGs comparing adjacent postpubertal female timepoints, and unlike the earlier timepoints, these DEGs were observed only in females (PD32 vs. PD37) (Fig. 2A). These DEGs included several downregulated puberty-relevant neuropeptides, including *Tacr1* [64] and *Sst* [65]. Upregulated DEGs included genes encoding transcriptional regulators involved in the hypothalamus-pituitary-gonadal axis (e.g., *Gnrh*, *Lhb*, *Ar*, and *Pgr*) such as *Cited2* [66], *Fgfr2* [61, 67], *Lcor* [68], and *Sp1* [69] (Supplementary Figure S3A, B). Interestingly, 23 of these upregulated DEGs are also downregulated (i.e., PD12 > PD22) before puberty in females (PD32 vs. PD37; 23 genes, FDR-adjusted p-value = 5.06 × 10^− 12^), representing a set of genes whose expression may be suppressed during puberty in female mice. This pattern is not found in male mice.

Pathway analysis of these overlapping genes enriched for “transcriptional co-repression activity” (*Skil*, *Wwtr1*, *Cited2*) (3 genes, FDR-adjusted p-value = 0.044) and pathways involved in cell and tissue development (Supplementary Figure S3C, Supplementary Figure S4). In contrast, we found eight upregulated DEGs before puberty (PD12 vs. PD22) enriched for the “response to peptide hormone” gene ontology (8 genes, FDR-adjusted p-value < 0.01). These genes include *Agrp*, which regulates pubertal activation (Supplementary Figure S3) [70]. Together, we identified a set of transcriptional repressors being suppressed during puberty, paired with hormonal genes being activated during puberty in female mice, reflecting previous literature [71, 72].

We only identified 2 and 12 DEGs between PD22 and PD27 and 12 and 32 genes between PD27 and PD32 in males and females, respectively. While few DEGs were identified, it is notable that 5 of the 6 DEGs found in males between PD27 and PD32 were puberty-relevant neuropeptides, including downregulated cholecystokinin (*Cck*) [73] and upregulated CART peptidase (*Cartpt*) [74, 75], pro-melanin concentrating hormone (*Pmch*) [76], orexin (*Hcrt*) [77, 78], and proopiomelanocortin (*Pomc*) genes [79] were upregulated (Figs. 1C and 2C and D). *Hcrt* [77, 78], oxytocin (*Oxt*) [80, 81], and *Axl* [82] are all differentially expressed in female mice and peak in expression at PD27 where we observe vaginal opening (PD22 < PD27 or PD27 > PD32; Fig. 2). Together, while the number of DEGs detected during puberty was lower than those detected before or after puberty, many of the peri-pubertal genes have previously been linked to pubertal regulation.

While we found developmental gene expression trajectories specific to female mice (Fig. 2A, Supplementary Figures S3, S4), we did not find many DEGs when performing pairwise differential expression between sexes at each timepoint. Across all timepoints, we found 41 male biased and 22 female biased genes, with almost half of these sex-biased mapping to a sex chromosome (chrX = 5, chrY = 21) (Supplementary Figure S5A, B). While there are fewer sex differences than DEGs across timepoints, four puberty-relevant genes are sex-biased. Specifically, *Tcf7l2* [83, 84] is female-biased at PD27, and *Etnppl*, *Cryab* [85], and *Hcrt* [77, 78] are male-biased at PD32 (Supplementary Figure S5C). Taken together, our pairwise differential analysis of age and sex individually identified expected gene developmental and hormonal gene expression patterns. However, analyzing each sex individually was insufficient to capture a dynamic signature of pubertal regulation when analyzing gene expression in the bulk hypothalamus.

### Genes expressing metabolic and reproductive neuropeptides display an age-by-sex interaction in gene expression along the pubertal transition

**Fig. 3:**
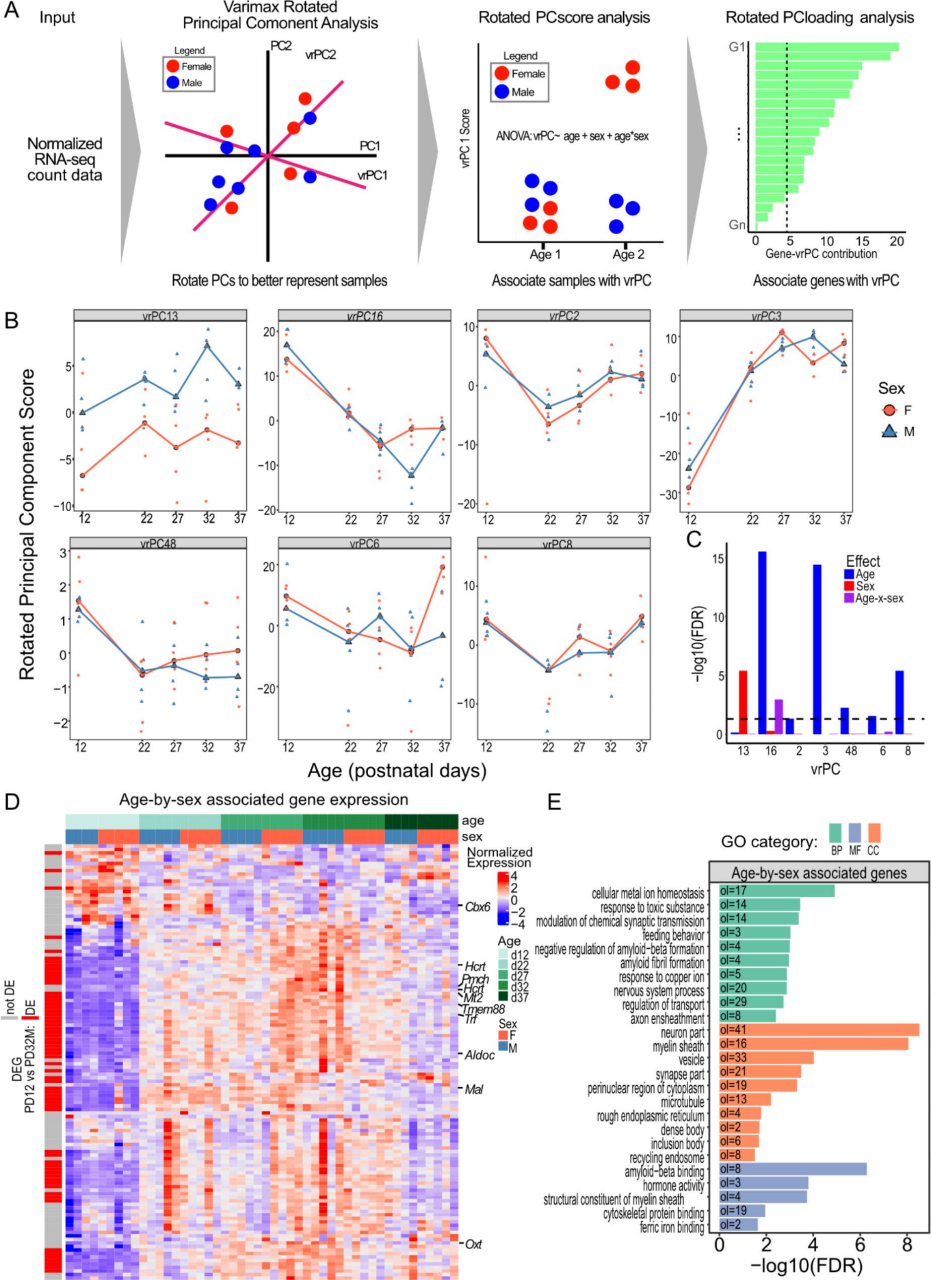
Evaluation of varimax-rotated principal component analysis revealed genes involved in sex-by-age interactions. **A**) Schematic of how varimax rotated PCA is applied to our data. **B**) Distribution of scores for enriched vrPCs. Male samples are triangles and blue lines, and female samples are circles and red lines. vrPC16 is highlighted because the genes associated with this vrPC are focused on for the rest of this study. **C**) Barplot showing the association between each significant varimax rotated PC (vrPC), age, and sex. The X-axis shows vrPCs whose scores are associated with age, sex, or an age-by-sex interaction (7/48 total vrPCs). Red bars show the significance of sex, blue bars show the significance of age, and purple bars show the significance of an age-by-sex interaction. **D**) Heatmap of the gene expression patterns of genes associated with vrPC16. Each row is a gene, and each column is a sample. The heatmap is populated by the log2-RUV-seq normalized gene expression of each gene. Rows are annotated by whether the gene displays pairwise expression in at least one pairwise timepoint. Columns are annotated by age and sex. **E**) Barplot of enriched pathways derived from genes strongly associated with rotated PC 16. Barplots show the -log10(FDR-adjusted P-value) of enrichment. Green bars represent pathways deriving from gene-ontology biology pathways, red bars represent pathways deriving from gene-ontology cellular components, and blue bars represent pathways deriving from gene-ontology molecular functions

Traditional differential expression analysis across timepoints and within each sex was able to capture many of the broad developmental processes occurring during postnatal development. However, it was unable to capture gene expression signatures involved in pubertal regulation. We aimed to combine all our gene expression data into a model that allowed us to investigate groups of genes whose developmental trajectories were offset or divergent between sexes. To do this, we leveraged the varimax rotated principal component analysis (vrPCA) [86] (Fig. 3A, See Materials and Methods for details), which is a technique used to improve the interpretation of principal components (Fig. 1D).

In total, we identified 129 age-by-sex associated genes, 66 of which were differentially expressed between PD12 and PD22 in males or females (Fig. 3). Interestingly, the four genes with the strongest association with an age-by-sex interaction based on their vrPC loading are all hormone-producing genes that have been linked to pubertal regulation or dynamics: *Pmch*, *Hcrt*, *Oxt*, and *Trf* [76, 77, 81, 87] (Fig. 3D). While these genes with top loadings shared similar expression patterns (i.e., an increase in gene expression from PD12-PD27 in both sexes before diverging by sex), 21 genes decrease in gene expression before diverging by sex (Fig. 3D). This includes an established puberty-regulator *Cbx6* [71], a member of the Polycomb repressive complex. We observed that three PD12 samples had different vrPC scores and expression patterns of genes with the top loadings, but removing these samples and repeating the analysis identified the same vrPC, showing that they are not driving the observed age-by-sex expression pattern (Supplementary Figure S6). Pathway enrichment of age-by-sex associated genes were enriched for hormone activity (precision = 0.100, FDR = 1.60 × 10^− 4^), negative regulation and transmission of nerve impulse (precision = 0.167, FDR = 0.0248), and neuron and oligodendrocyte development pathways, including “neuron part” (precision = 0.339, FDR = 2.99 × 10^− 9^) and “myelin sheath” (precision = 0.132, FDR = 8.82 × 10^− 8^) (Fig. 3E).

We paired our pathway enrichments of these age-by-sex associated genes with enrichments against a set of human RNA-seq DE experiments stored within the differential expression enrichment tool (DEET) and database. Here, the age-by-sex associated genes enriched for human DEG comparisons influencing glial cell growth in the TCGA database and neuronal-related pathologies in the GTEx database (Supplementary Figure S7A). Interestingly, these age-by-sex associated genes also enriched for many relevant comparisons in the hypothalamus, including age and body mass index (BMI) from the GTEx database (Supplementary Figure S7B). The genes driving the enrichment of these hypothalamus comparisons included puberty-relevant hormonal neuropeptides with a high vrPC16 loading, namely *OXT*,* AVP*,* HCRT*, and *PMCH* (Supplementary Figure S7C, D).

Recently, spatially resolved single-cell transcriptomics have been performed along the pubertal transition of the female rat arcuate nucleus [4]. They identified three gene-expression modules associated with the pubertal transition. Broadly, they categorized genes associated with these modules as: module (1) glial cell enhancement and neuron proliferation in response to estradiol, module (2) hormone secretion, and module (3) neuronal differentiation and signal transmission [4]. The age-by-sex associated genes we identified were over-represented in all three modules (module 1: p-value = 2.23 × 10^− 13^, odds-ratio = 6.80, genes = 29; module 2: p-value = 0.0506, odds-ratio = 1.97, genes = 11; module 3: *p* = 4.11 × 10^− 11^, odds-ratio = 4.22, genes = 38). Together, genes associated with an age-by-sex interaction across puberty are over-represented in pathways and cell-type trajectories driving hypothalamic hormonal activity, neuronal development, and oligodendrocyte development, reflecting a gene expression signature linked to pubertal regulation.

### Cellular composition of the postnatal hypothalamus

**Fig. 4:**
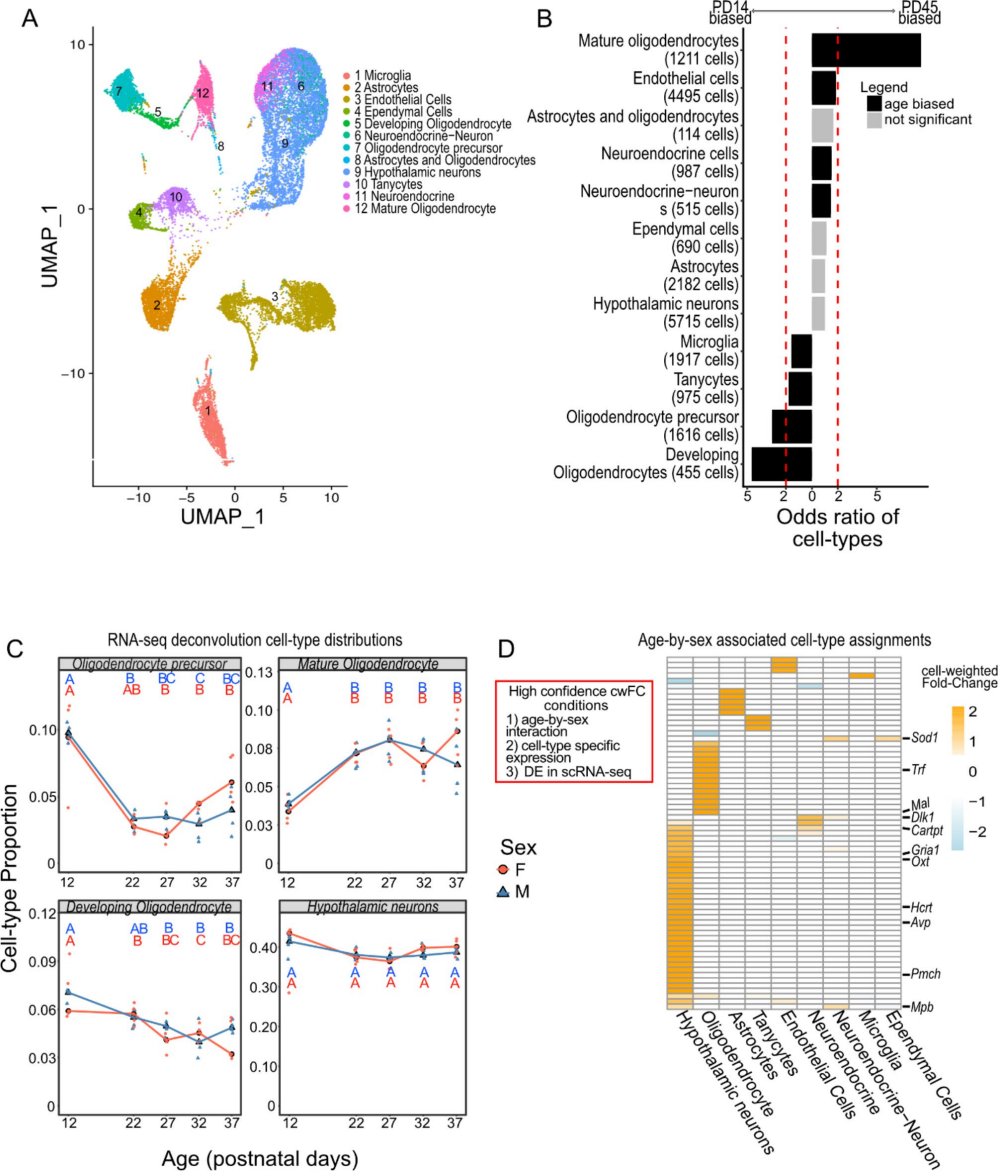
Cell-type specific gene expression across the developing hypothalamus. **A**) Lower-dimension representation of scRNA-seq data in the PD14 and PD45 mouse hypothalamus with the Uniform Manifold Approximation and Projection (UMAP). Cell labels were identified using a mixture of labels provided by Kim et al., 2020 and unsupervised clustering. **B**) Distribution of cell proportions estimated from RNA-seq deconvolution at each age and time-point. The X-axis is age, and the Y-axis is estimated cell-type proportions. Red lines and circles represent female samples, while blue lines and triangles represent male samples. Letters represent significance using a Tukey post hoc test after identifying differences in cell-type proportion with ANOVA. **C**) Barplot of cell-type proportion differences within each cluster (Fisher’s exact-test). Red bars designate a fold-change of two between ages. Each column is a cell-type with the number of DEGs mapping to that cell-type in brackets. **D**) Heatmap of gene-normalized cell-weighted fold-changes (cwFold-changes) of the 129 age-by-sex associated genes and are DE in the complementary direction in the scRNA-seq data

The hypothalamus exhibits considerable cellular heterogeneity reflecting its multimodal functions [2, 20]. To characterize the cell type-specific underpinnings of pubertal development in the hypothalamus, we integrated scRNA-seq in the hypothalamus with our temporal bulk RNA-seq. We leveraged data from Kim et al., 2020, which contained scRNA-seq from the mouse hypothalamus before and after puberty (PD14 and PD45) [3]. We incorporated the cell-type labels provided by Kim et al., 2020 (hypothalamic neurons, oligodendrocytes, tanycytes, ependymal cells, astrocytes, microglia, and endothelial cells) [3] with cell-type identification analysis of clusters measured with Seurat [38] (see Materials and Methods for Details). Our cluster analysis further subdivided oligodendrocytes into oligodendrocyte precursor cells (OPCs), developing oligodendrocytes DOs, and mature oligodendrocytes (MOs). It also subdivided neurons into neurons and neuroendocrine cells (Fig. 4).

We first investigated hypothalamic cell-type proportion dynamics across pubertal timepoints. When investigating the scRNA-seq data alone, we found that oligodendrocytes were the most dynamic cell types across puberty (Fig. 4B), with MOs increasing in proportion over time (PD14 < PD45) (Bonferroni-adjusted p-value = 1.90 × 10^− 106^, fold-change = 8.43), and OPCs (Bonferroni-adjusted p-value = 3.94 × 10^− 99^, fold-change = -3.07) and DOs (Bonferroni adjusted p-value = 8.18 × 10^− 56^, fold-change = -4.65) decreasing in proportion over time (PD14 > PD45). There was also a lesser but significant increase in endothelial (Bonferroni-adjusted p-value = 1.09 × 10^− 54^, fold-change = 1.856) and neuroendocrine cell (Bonferroni adjusted p-value = 4.81 × 10^− 7^, fold-change = 1.52) proportions over time.

Next, we used estimated hypothalamic cell-type proportions in our bulk RNA-seq data and RNA-seq deconvolution, mapping cell-type proportion changes across our developmental trajectory. Benchmarking RNA-seq deconvolution in the hypothalamus is important because it has both highly similar cell types (e.g., neuron vs. neuroendocrine) and highly distinct cell types (neuron vs. endothelial cell) amongst its many total cell types. To find the most reliable RNA-seq deconvolution tool in our system, we compared the cell-type proportions of nine different RNA-seq deconvolution tools to the scRNA-seq data (See Materials and Methods for Details). We found that the MuSiC-NNLS tool [7] was the most accurate method, a method that has previously performed well on brain tissue [88] (Supplementary Table S2). As in the scRNA-seq data, we found that MOs increase in cell-type proportion until puberty, and OPCs (p-value = 3.68 × 10^− 11^) and DOs decrease in cell-type proportion until puberty (Fig. 4C, Supplementary Figure S8). These results further support our results suggesting that hypothalamic oligodendrocytes expand from OPCs into MOs during puberty.

### Spatial composition of oligodendrocytes in the postnatal hypothalamus

To gain insight into where the inferred expansion of oligodendrocytes is occurring in the hypothalamus, we interrogated the Allen Brain Cell Atlas [59] (see Materials and Methods for details). The Allen Brain Cell Atlas (ABCA) includes over 3.5 million spatially resolved cells across the adult C57BL6/J mouse brain [59]. Of these cells, ~ 38,000 belong to the hypothalamus, with ~ 6000 of the hypothalamus-assigned cells being oligodendrocytes (Supplementary Fig. 9A-B). We found the lateral hypothalamic area (LHA) and the zona incerta (ZI) to be the regions most over-represented in oligodendrocytes (Supplementary Fig. 9C, D,E, F). The LHA is predominantly characterized by the orexigenic neurons [89], defined in part by the expression of the orexin-encoding gene *Hcrt*, which we identified as one of the genes with an age-by-sex interaction based on its vrPC loading.

Next, we re-analyzed spatially resolved gene expression of the arcuate nucleus in pre-pubertal (PD25), peripubertal (PD35), and post-pubertal (PD45) female rats published in Zhou et al., 2022 [4, 90] (see Materials and Methods for details). The PD25 timepoint the rat approximates our PD21 pre-pubertal timepoint in the mouse [4]. Therefore these spatially-resolved oligodendrocyte proportion changes reflect the pubertal timepoints in our data, rather than the early PD12-PD22 timepoint. These data allow for the interrogation of oligodendrocyte expansion spanning the pubertal transition.

Like in the mouse, we found most oligodendrocytes in the lateral hypothalamic (LH) area of the arcuate nucleus. In addition, the pre and post-pubertal experiments allowed us to detect a ~ 7-fold depletion in OPC-containing spots from PD25 and PD35 and no difference between PD35 and PD45 in the rat LH, (Supplementary Fig. 10C), reflecting the decrease in OPCs found in our data (Fig. 4B, C). Each “spot” in the LH is saturated with MOs at each timepoint (i.e., MO confidence is > 95% at each timepoint), preventing us from asking whether MO proportions are increasing during puberty in the LH. Lastly, these data include a region of the thalamus (i.e., the paraterete nucleus), which displayed a consistent decrease in MOs between PD25, PD35 and PD45, which shows that our findings do not reflect a secular increase in MOs in the brain.

### Age-by-sex associated transcriptional dynamics map to genes involved in neuropeptide activation and oligodendrocyte maturation

We next used these scRNA-seq data to assign age-by-sex associated genes to their cell type of origin using scMappR [5], using both the PD14 and PD45 timepoints in the scRNA-seq data [3]. Overlapping the 129 cell type-specific age-by-sex associated genes with cell type-specific DEGs from the scRNA-seq data [3, 47] (PD14 vs. PD45) yielded a set of high-confidence cell-type specific genes (*n* = 67), whose gene expression patterns are conditional on both age and sex (Fig. 4D). Of the four highest confidence genes with the top age-by-sex loadings, three mapped to neurons and neuroendocrine cells, namely *Pmch*, *Hcrt*, and *Oxt*, while *Trf* mapped to oligodendrocytes. Previous work showed that *Trf* acts as a cofactor for iron in myelination [91]. Additionally, *Dlk1* and *Gria1* mapped to neuroendocrine cells and neurons (Fig. 4D, Supplementary Figure S11) and are implicated in pubertal disease and ovulation rate respectively [92, 93].

Next, we investigated whether the neuron- and neuroendocrine-mapping age-by-sex associated genes overlapped with translated mRNA in lepRb + neurons in the hypothalamus [37] using TRAP-seq (i.e., RNA-seq of ribosome-bound mRNA), because leptin is an activator of pubertal initiation [70, 94]. We found 21 of our neuron- and -neuroendocrine-mapping genes were enriched in lepRb + neurons (p-value = 4.33 × 10^− 9^, odds ratio = 5.96) (Supplementary Figure S12). These genes included *Cartpt*, *Dlk1*, and *Sod1*, which have all been previously shown to influence pubertal timing or fertility [74, 95–97].

Next, we aimed to increase the specificity of our cell-type specific gene expression analysis by identifying neuronally-expressed genes with an age-by-sex interaction in gene expression before assigning them to their neuronal subtype. Briefly, we used BayesPrism [6] to predict neuron-specific gene expression in our RNA-seq data. Then, we generated a signature matrix of neuronal subtypes in the scRNA-seq data used in this study [3] before repeating the same varimax rotation analysis (see Materials and Methods for details). We identified one neuronal vrPC with an age-by-sex interaction (neuron-vrPC 11) (Supplementary Figure S13A). Neuron-vrPC 11 shared the distribution of its PC score with the unadjusted age-by-sex associated principal component (i.e., vrPC 16) and 45/77 (58.4) of the genes associated with neuron-vrPC 11 were found in the unadjusted age-by-sex associated gene set. We found several of the genes unique to the neuron-vrPC 11 were previously reported to be involved in pubertal regulation or disorders that impact the normal development of secondary sex characteristics, such as *Th* (encoding thyroid hormone) [98], *Nrxn1* [99], *Cpe* [100], and *Xist* [101] (Supplementary Figure S14C).

Next, we used scMappR [5] to assign these neuron-vrPC 11 associated genes into their neuronal subtype of origin (See Materials and Methods for details). Most of these genes were assigned to *Cck*-*Cartpt* + GABAergic neurons, *Oxt* + *Avp* + *Pdyn* + neurons, or neuroendocrine cells (Supplementary Figure S14). Additionally, the polycomb repressive complex gene *Cbx6* is only assigned to *Cck*-*Cartpt* + GABAergic neurons and is decreasing in expression (Supplementary Figure S15).

### Lineage reconstruction of oligodendrocytes during postnatal mouse hypothalamus development

**Fig. 5:**
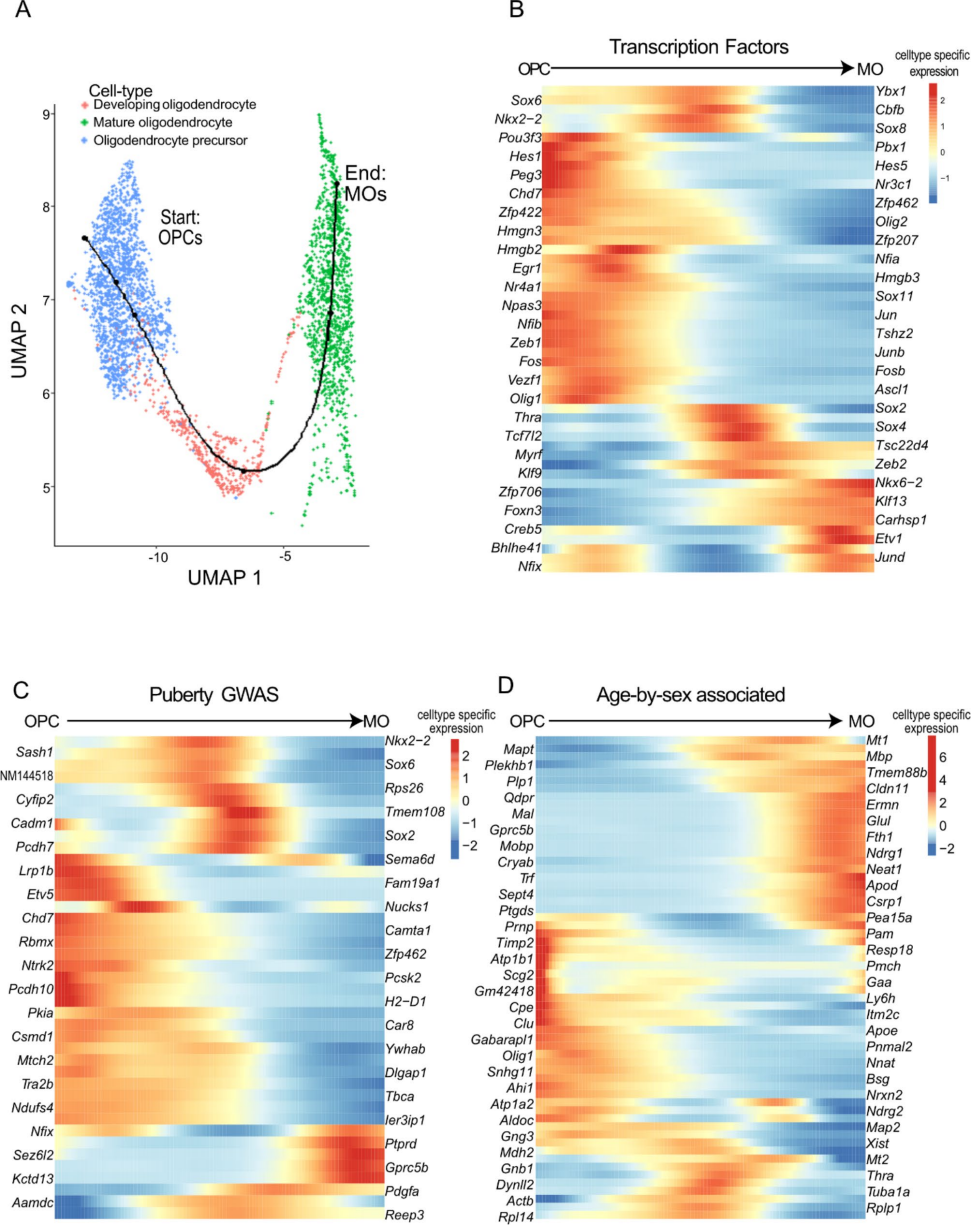
Pseudotime of hypothalamic oligodendrocyte development. **A**) Lower-dimension representation of oligodendrocyte scRNA-seq data in the PD14 and PD45 mouse hypothalamus with the Uniform Manifold Approximation and Projection (UMAP) overlaid with the pseudotime trajectory identified with Slingshot. Points are cells coloured by cell-type. and the line is the plotted pseudotime trajectory measured with Slingshot, starting with OPCs and plotted with the tradeSeq R package. **B**) Heatmap of transcription factors associated with pseudotime. **C**) Heatmap of puberty-associated GWAS genes associated with pseudotime. **D**) Heatmap of oligodendrocyte-mapped age-by-sex associated genes associated with pseudotime. For a gene to be included, it must be associated with an age-by-sex interaction (i.e., varimax 16), mapping to oligodendrocyte precursor cells, developing oligodendrocytes, or mature oligodendrocytes with scMappR, and associate with pseudotime. For **B**-**D**, rows are genes associated with pseudotime. Columns are portions of the pseudotime trajectory blocked into 200 smoothers using tradeSeq. Heat is measured by scaling the predicted smoothers with the scale function in R

We observed an active transition of oligodendrocytes from OPCs into MOs in the postnatal hypothalamus (Figs. 2B and D and 4), and detected clear manifold from OPCs to DOs and MOs in the reprocessed scRNA-seq data (Fig. 4A). By using Slingshot, a bioinformatic package that identifies cellular lineages across cell types [53], we measured a pseudotime trajectory from OPCs to MOs (Fig. 5A). By using tradeSeq [54], we could map cell-type specific gene expression to the cellular trajectory measured using Slingshot [53] (see Materials and Methods for details).

Overall, we found 1294 genes with a dynamic gene expression pattern along the OPC-to-MO cellular trajectory. We overlapped these genes with transcription factors (Fig. 5B), genes implicated in age of menarche or voice breaking from genome-wide association study [56, 98] (Fig. 5C), and with our oligodendrocyte-expressed age-by-sex associated genes (Fig. 5D). Transcription factors associated with oligodendrocyte development were most prominently expressed in developing oligodendrocytes (5B), where we found genes involved in thyroid hormone response (*Nkx2-1* and *Thra*) and cell differentiation and development (*Sox2*, *Tcf7l2*, *Egr1*, *Hes1*) (Fig. 5B, C). Next, we found that these genes associated with oligodendrocyte development were over-represented for both puberty-related GWAS hits genes (FDR = 4.6 × 10^− 3^_,_ 27 genes) and oligodendrocyte-expressed age-by-sex associated genes (FDR = 4.6 × 10^− 3^, 58 genes).

Of the 58 genes displaying an age-by-sex interaction and gene expression and an association with oligodendrocyte development, we found an even distribution of genes mapping to OPCs, DOs, and MOs (Fig. 5D). These genes included core oligodendrocyte stage-specific regulated proteins such as *Mbp*, *Mobp*, *Mal*, and *Olig1* [45], puberty and HPA-linked *Thra*, genes encoding the melatonin receptors *Mt1* and *Mt2*, and *Pmch* (Fig. 5D). Three of these genes, namely *Sox2*, *Chd7*, and *Stub1*, can lead to HH in humans [60]. *Chd7* works with *Sox10* to promote myelination by co-occupying and promoting the expression of myelinogenic genes [99], and *Stub1* has a less studied role in oligodendrocytes. *Sox2* plays an important role in cellular differentiation, proper myelination, hypothalamic-pituitary-gonadal axis development, and many other development processes [100, 101]. Lastly, two transcriptional regulators whose mutations lead to both HH and hypomyelination are *Polr3a* and *Polr3b* [60, 102]. We found that other members of the polymerase 3 complex, namely *Polr3e* and *Polr3h*, have dynamic gene expression patterns along the cellular trajectory of OPCs to MOs (Supplementary Figure S15). Whether these findings link oligodendrocyte development in the hypothalamus to pubertal progression remains to be seen.

## Discussion

The regulation of puberty (onset and progression) is a dynamic, non-linear process that is also sex-biased in its initiation, regulation, and manifestation. By integrating bulk RNA-seq spanning 5 timepoints in male and female mice with publicly available scRNA-seq data of pre- (PD14) and postpubertal (PD45) mice [3], we identified gene expression signatures and genes, some of which are known to be involved in pubertal regulation and other candidates whose role remains to be seen.

Differential gene expression in male and female mice during puberty (i.e., PD22-PD27, PD27-PD32) included genes coding for pubertal and metabolic hormones and neuropeptides, namely *Oxt*, *Hcrt*, *Cartpt*, *Avp*, *Cck*, *Pmch*, and *Pomc* [73, 74, 76, 77, 79, 81, 103, 104]. Han et al., 2020 also identified many of these same genes (including *Oxt*, *Hcrt*, *Avp*, *Cartpt*, *Pomc*,* Pmch*) as differentially expressed in the arcuate nucleus and/or premammillary nucleus of the hypothalamus in a leptin-inducible transgenic model of pubertal activation in female mice [85]. While many of the individual peripubertal DEGs identified in this study have been previously implicated in pubertal regulation, the number of detected DEGs was low compared to over the ~ 1,000 DEGs observed prepuberty (PD12 vs. PD22), which in both male and female mice were enriched for pathways related to the cellular organization of the hypothalamus.

To better incorporate sex as a variable, we turned to an established varimax PCA approach [25] that allowed us to identify genes whose variation is related to both age and sex. This analysis revealed 129 genes with age by sex interactions, including the puberty relevant DEGs *Oxt*, *Hcrt*, *Cartpt*, *Avp*, *and Pmch*. Three potentially puberty-relevant, age-by-sex associated genes that could be assigned to neuronal cell types were *Gria1*, *Dlk1*, and *Cartpt*. Recent studies in female rats show that *Gria1*, along with a network of genes implicated in the epigenetic control of puberty, is under the shared regulation of *Kdm6b* at puberty in the hypothalamus [93]. Loss of function variations in *Dlk1* are associated with CPP, and common variants near *Dlk1* are associated with age of menarche [95, 103, 105]. *Dlk1*’s ability to regulate Notch signaling has been a proposed mechanism to control pubertal timing [105]. *Cartpt* plays a core role in the function of CART neurons, a neuronal subtype that receives signals from leptin and alters pubertal timing in female mice [74]. *Cartpt* was also found by Han et al. to be differentially expressed in the hypothalamus of leptin deficient mice given exogenous leptin to initiate puberty [85]. Lastly, our integrative analyses of bulk and single cell RNA-seq data that assigned age-by-sex associated genes to neuronal subtypes identified 21 genes mapping to CART neurons (Supplementary Figure S14). *Cbx6*, a component of the Polycomb repressive complex (PRC), mapped to these CART neurons and displayed an inverse gene expression pattern to *Cartpt*. Given the PRCs critical role in regulating kisspeptin neurons during pubertal activation [71, 72], the sex-dependent downregulation of *Cbx6* and upregulation of *Cartpt* during puberty warrants further investigation.

Previous studies reported that oligodendrocyte maturation and myelination can continue into adolescence [106–108]. However, the magnitude of oligodendrocyte expansion and its role in regulating hypothalamic hormones is not well understood [109]. When we integrated our bulk RNA-seq data with publicly available scRNA-seq data [3] in the hypothalamus, we inferred a substantial expansion of OPCs into MOs before and during puberty (Fig. 4B, C). Furthermore, we observed that age-by-sex associated genes obtained by our vrPC analysis were over-represented in the set of dynamic genes across oligodendrocyte expansion (Fig. 5A, D). By analyzing the mouse Allen Brain Cell Atlas [59] and publicly available spatial transcriptomic data in the adult rat hypothalamus [90], we observed that the major site of hypothalamic maps to the lateral hypothalamus (LH) (Supplementary Figure S9, S10). The lateral hypothalamic area plays an important role in feeding, arousal, pain, and body temperature [110]. The LH also contains a high density of orexinergic (*Hcrt* expressing), melanin concentrating-hormone secreting (*Pmch* expressing) and CART (*Cartpt*) expressing neurons. Each of these LH-enriched cell-types contains key marker genes displaying an age-by-sex interaction, and all of these cell-types have been shown to regulate pubertal development [74, 76, 78]. The shared localization and expression pattern of these genes and our observed hypothalamic oligodendrocytes leads us to speculate that these oligodendrocytes are supporting orexinergic, melanin concentrating-hormone secreting, and CART neurons during puberty in a sex-specific manner. Whether the hypothalamic oligodendrocyte expansion and the factors that control it play a supporting role in establishing puberty remains to be seen. Recently, Steadman et al., 2020 developed an inducible *Mrf* knockout that blocks OPC expansion into MOs [111]. Adapting this model to be hypothalamus-specific could, in principle, test if oligodendrocyte expansion influences pubertal initiation.

One important limitation of our study is that we investigated the entire hypothalamus rather than micro-dissecting the hypothalamus into subregions. The advantage of our study design, which opted for low cellular resolution and high biological replicates (i.e., *N* = 48 in one experimental batch), is that it allowed us to study male and female mice while maximizing the number of timepoints and biological replicates and aligned with existing single cell transcriptomic datasets performed on the entire hypothalamus. However it is worth noting that although our study incorporated published, high quality, scRNA-seq from postnatal timepoints highly relevant to our study (PD14 and PD45; [3]), this study was not designed to study puberty. The scRNA-seq data we used was generated using two different strains and did not control for sex (PD14 mice were CD1 (male and female), and PD45 mice were C57BL/6J (male). Furthermore, while we could provide cellular resolution on our bulk gene expression data, could not capture the gene expression dynamics of rare cell-types within the hypothalamus. For example, many cell-types that play the most important role in regulating puberty (e.g., Gnrh neurons, KNDy neurons) are rare and require prior cell-sorting for surface markers to observe them in a typical scRNA-seq experiment [79].

Spatial resolution is important to understand hypothalamic function [112, 113], however neither of the bulk RNA-seq or scRNA-seq data in this study is spatially resolved. We used publicly available spatial data to infer the spatial distributions of oligodendrocytes in our data, however, due to our data being bulk and not spatially resolved, we still cannot detect developmental spatial dynamics specific to subregions of the hypothalamus. Similar to how our integrative bulk/single cell RNA-seeq analysis would not provide enough resolution to study Gnrh and KNDy neurons, the recently reported increase in *SEMA6A* expressing oligodendrocytes that is relevant for regulating Gnrh neurons in the median eminence could not be detected in our analyses [114].

Another limitation of our study is that the biological inferences that we make about the gene expression dynamics are based on discoveries made by previous work, rather than our own functional assays. As such, our findings are correlative rather than causal in nature. This is inherent to studies that exclusively rely on gene expression. Nonetheless gene expression profiling by RNA-seq has been a consistently reliable method to aid in hypothesis generation and downstream biological discovery. A recent tour-de-force study looking at gene regulatory networks underlying sexually dimorphic neural circuits utilized microdissection of specific brain regions in conjunction with sensitive genomics approaches to map the genome wide binding of the estrogen receptor to target genes [115]. Such empirical transcription factor-chromatin assays (and other cell-type specific mouse transgenic tools such as translating ribosome affinity purification followed by RNA-seq), will be essential for building gene regulatory networks related to pubertal timing.

### Perspectives and significance

Despite intense study over several decades, the mechanisms controlling the timing of puberty are still unclear. The hypothalamus central to our understanding of how pubertal timing is controlled. Finding the genes that control pubertal timing will provide direct insight into how the hypothalamus integrates environmental signals such as those derived from diet, psychosocial stress, and photoperiod [109]. Our study found that acquiring a puberty-driven gene expression signature was possible in the hypothalamus when profiling multiple timepoints in surrounding puberty in males and females. By taking care to make these results easily accessible, we intend for this work to be a useful resource for investigating post-natal development in the mouse hypothalamus. Our integrative analyses allowed us to identify biologically interpretable gene expression patterns from a complex process and heterogeneous tissue. Subspace simplification techniques (i.e., a varimax rotation simplifying a principal component space) are less common in bulk RNA-seq analysis than differential gene expression and gene correlation network analyses. We propose that these subspace simplification techniques are valuable when investigating complex biological processes (e.g., multiple sexes, a treatment, and/or timepoints) a multicellular RNA-seq samples (i.e., bulk RNA-seq) provided sufficient biological samples.

## Conclusions

We found that cell type- and sex-aware transcriptomic dynamics in the pubertal hypothalamus are associated with well-established neuropeptide activation and regulation, as well as potentially relevant genes including *Hcrt*, *Oxt*, *Dlk1*, *Gria1*,* and Cartpt*. We inferred that oligodendrocyte expansion occurs in the hypothalamus prior to and throughout pubertal initiation and that many genes associated with this oligodendrocyte expansion relate to pubertal timing and regulation. Our data and interactive Shiny App will allow researchers to visualize the transcriptionally dynamic genes in the hypothalamus and pituitary gland, providing a baseline in postnatal gene expression for the broader scientific community.

## Electronic supplementary material

Below is the link to the electronic supplementary material.

Supplementary Material 1

Supplementary Material 2

Supplementary Material 3

Supplementary Material 4

Supplementary Material 5

Supplementary Material 6

Supplementary Material 7

Supplementary Material 8

## Data Availability

RNA-seq data generated for this current study is available at ArrayExpress (E-MTAB-12340). A stable, downloadable instance of our Shiny App is stored on Zenodo (DOI: 10.5281/zenodo.10899706). Code and data to reproduce the figures in this study is stored on FigShare (DOI: 10.6084/m9.figshare.25517503).
