# Supplementary material for: Age, sex, and cell type-resolved hypothalamic gene expression across the pubertal transition in mice
Source: Biol Sex Differ. 2024 Oct 24;15:83. doi: 10.1186/s13293-024-00661-9 (PMC11515584; doi:10.1186/s13293-024-00661-9)
Supplement: Supplementary file 1 — Supplementary Material 1 [file 13293_2024_661_MOESM1_ESM.docx]

# Age, sex, and cell type-resolved hypothalamic gene expression across the pubertal transition in mice

Dustin J. Sokolowski^1,2^, Huayun Hou^1^, Kyoko E. Yuki^1^, Anna Roy^1^, Cadia Chan^1,2,3^, Wendy Choi^1,2,3^, Mariela Faykoo-Martinez^1,5^, Matt Hudson^1,2^, Christina Corre^1^, Liis Uusküla-Reimand^1^, Anna Goldenberg^1,8,9,10^, Mark R. Palmert^1,6,7,11^, Michael D. Wilson^1,2,#^

(1) Genetics and Genome Biology, SickKids Research Institute, Toronto, ON, Canada

(2) Department of Molecular Genetics, University of Toronto, Toronto, ON, Canada

(3) Developmental and Stem Cell Biology, The Hospital for Sick Children, Toronto, ON, Canada

(4) Donnelly Centre for Cellular & Biomolecular Research, Toronto, ON, Canada

(5) Department of Cell and Systems Biology, University of Toronto, Toronto, ON, Canada

(6) Division of Endocrinology, The Hospital for Sick Children, Toronto, ON, Canada

(7) Departments of Pediatrics and Physiology, University of Toronto, Toronto, ON, Canada

(8) Department of Computer Science, University of Toronto, Toronto, ON, Canada

(9) Vector Institute, Toronto, ON, Canada

(10) CIFAR, Toronto, ON, Canada

(11) Institute of Medical Science, University of Toronto, Toronto, ON, Canada

*corresponding author: michael.wilson@sickkids.ca

Author emails:

dustin.sokolowski@sickkids.ca

huayun.hou@sickkids.ca

kyoko.yuki@gmail.com

anna.roy@sickkids.ca

[cadia.chan@mail.utoronto.ca](mailto:cadia.chan@mail.utoronto.ca)

wendywy.choi@mail.utoronto.ca

m.faykoomartinez@mail.utoronto.ca

matt.hudson@sickkids.ca

calmorama@gmail.com

liis.uuskula-reimand@sickkids.ca

nyulik@gmail.com

mark.palmert@sickkids.ca

michael.wilson@sickkids.ca

**Table of Contents**

**Supplementary Figure S1**. Quality control metrics of our high-throughput mRNA-seq data. (Page 4).

**Supplementary Figure S2**. Summary of differentially expressed hypogonadotropic hypogonadism genes. (Page 5).

**Supplementary Figure S3**. Summary of differentially expressed genes (DEGs) between PD32 vs. PD37 in female mice. (Page 6).

**Supplementary Figure S4**. Summary of transcriptional co-repressors that decrease in expression during puberty and pubertal-relevant neuropeptides that increase in expression during puberty. (Page 8).

**Supplementary Figure S5**. Overview of sex differences across each pubertal timepoint. A) Volcano plots of sex differences at each time point. (Page 10).

**Supplementary Figure S6.** Identification of an age-by-sex varimax rotated principal component with variable PD12 samples removed (Page 12).

**Supplementary Figure S7**. Enrichment of age-by-sex associated genes against 3162 human RNA-seq comparisons stored in the DEET dataset. (Page 13).

**Supplementary Figure S8**. Distribution of cell-type proportions measured from MuSiC-NNLS in cell-types that were predicted to have >3% of the total sample. (Page 15).

**Supplementary Figure S9.** Distribution of mature oligodendrocytes across the adult mouse hypothalamus using the Allen Brain Cell Atlas (Page 17).

**Supplementary Figure S10.** Spatially resolved dynamics of oligodendrocyte precursor cells and mature oligodendrocytes across puberty in the female rat preoptic area (Page 19).

**Supplementary Figure S11**. Heatmap of gene-normalized cell-weighted fold-changes (cwFold-changes) of the 129 age-by-sex associated genes and are DE in the complementary direction in the scRNA-seq data. (Page 21).

**Supplementary Figure S12**. GeneMANIA plot of the 21 neuron- neuroendocrine-mapping age-by-sex associated genes that are detected as translated in hypothalamic LepRb+ neurons in Trap-seq from Allison et al., 2015. (Page 23).

**Supplementary Figure 13.** Evaluation of an age-by-sex enriched varimax rotated principal component in neuron-adjusted RNA-seq data (Page 24).

**Supplementary Figure 14.** Heatmap of gene-normalized cell-weighted fold-change (Fold-changes) of the 77 age-by-sex associated genes from neuron-adjusted RNA-seq data (Page 26).

**Supplementary Figure S15**. Pseudotime of hypothalamic oligodendrocyte development. Heatmap of RNA polymerase subunit genes associated with pseudotime. (Page 28).

**Supplementary Table S1**. Summary of RNA-seq sample quality and read mapping using Qualimap (Page 29).

**Supplementary Table S2**. Correlation and number of cell-types detected (>1% of the total population) between cell-type proportions from RNA-seq deconvolution and cell-type proportions from scRNA-seq data in the mouse hypothalamus (Page 30).


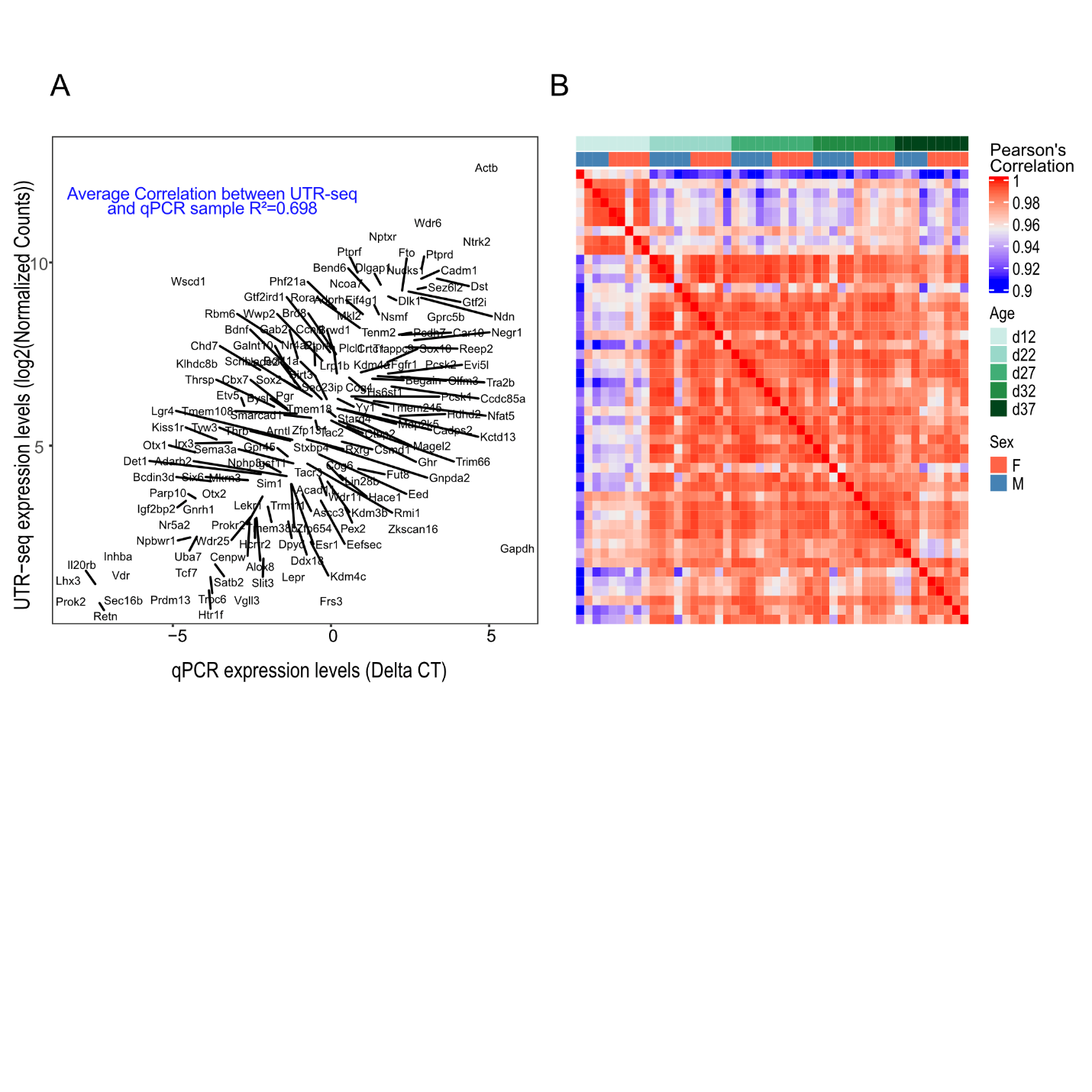


**Supplementary Figure S1. Quality control metrics of our high-throughput mRNA-seq data.** A) Correlation between UTR-seq and qPCR of 182 genes deriving from the same RNA. The X-axis represents each gene's average qPCR expression level across all 48 samples. The Y-axis is the average log2(RUV-seq)-normalized gene expression profiles across all 48 samples. B) Correlation heatmap of all UTR-seq samples, where all genes were included. Rows and columns are samples, and the heatmap is populated by Pearson’s correlation between them. Columns are annotated by age and sex and labeled by replicate number.


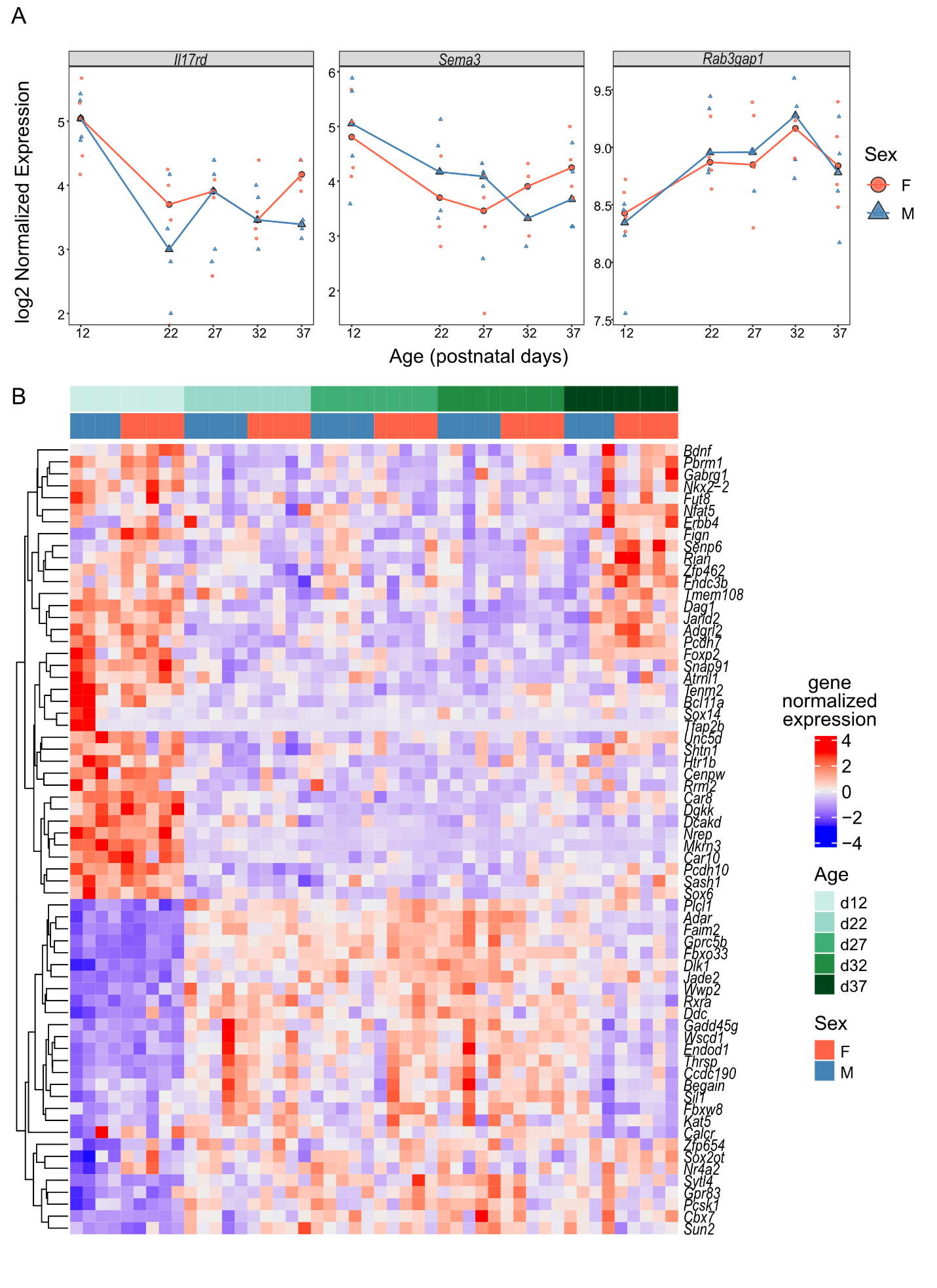


**Supplementary Figure S2. Summary of differentially expressed hypogonadotropic hypogonadism genes** A) Distribution of normalized counts from hypogonadotropic hypogonadism genes. The X-axis is age, and the Y-axis is log2-transformed RUVseq and ERCC-spike in normalized counts. Red lines and circles represent female samples, while blue lines and triangles represent male samples.
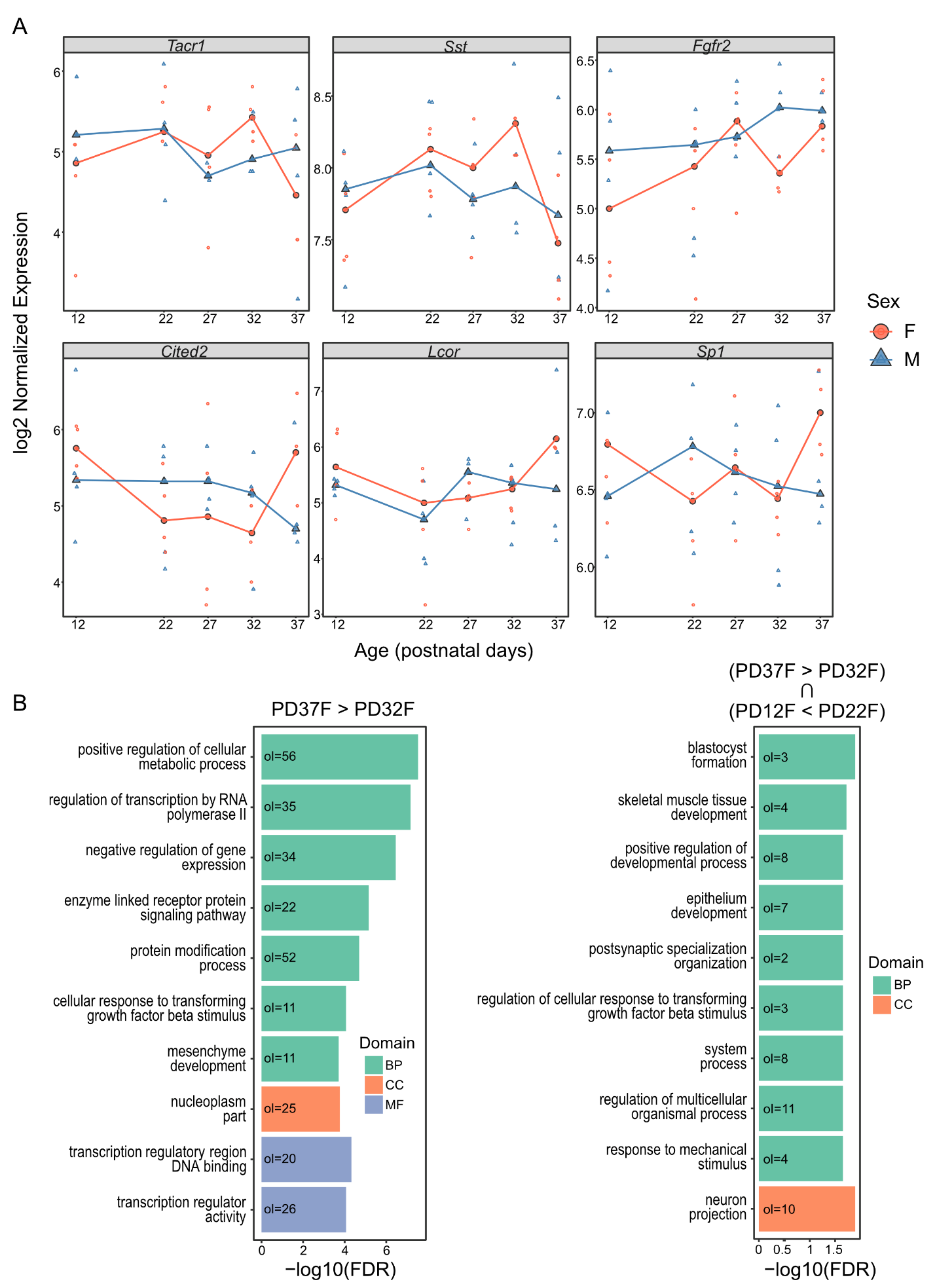


**Supplementary Figure S3. Summary of differentially expressed genes (DEGs) between PD32 vs. PD37 in female mice.** A) Distribution of normalized counts from hormone regulators/producers (*Tacr1*, *Sst*) and transcriptional regulators (*Fgfr2*, *Cited2*, *Lcor*, *Sp1*). The X-axis is age, and the Y-axis is log2-transformed RUVseq and ERCC-spike in normalized counts. Red lines and circles represent female samples, while blue lines and triangles represent male samples. B) Barplot of pathway enrichment of upregulated DEGs between PD37 vs. PD32 in female mice. C) Barplot of pathway enrichment of the overlap of upregulated DEGs between PD37 vs. PD32 in female mice and of downregulated DEGs between PD12 vs. PD22 in female mice.


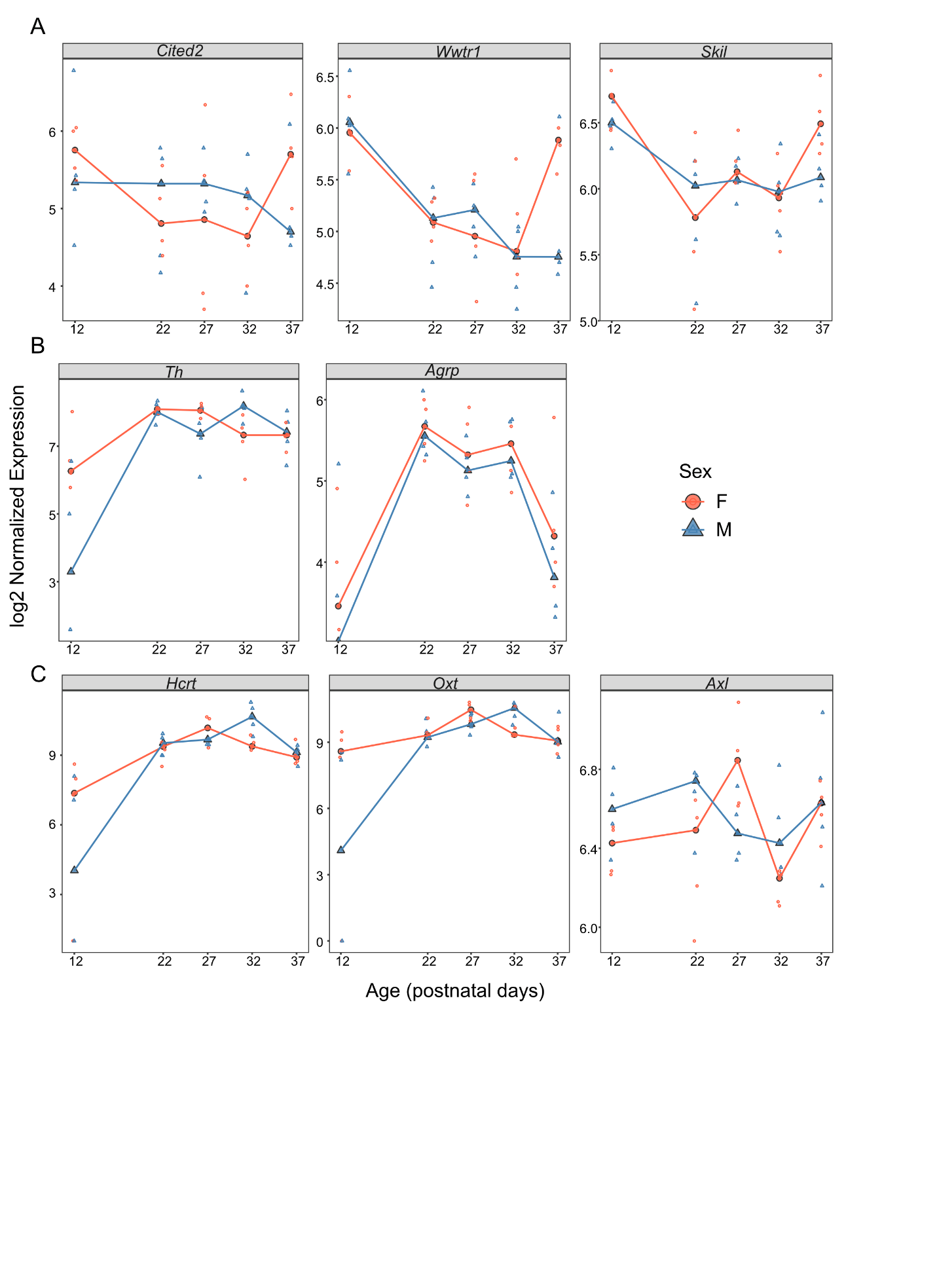


**Supplementary Figure S4. Summary of transcriptional co-repressors that decrease in expression during puberty and pubertal-relevant neuropeptides that increase in expression during puberty.** The X-axis is age, and the Y-axis is log2-transformed RUVseq and ERCC-spike in normalized counts. Red lines and circles represent female samples, while blue lines and triangles represent male samples. A) Transcriptional co-repressors that increase in expression between PD12 vs. PD22 in females and increase in expression between PD32 vs. PD37 in females. B) Direct pubertal regulators that increase in gene expression between PD12 vs. PD22 in females. C) Hormonal neuropeptide genes which peak in expression at PD27 in females, when half of the female mice have undergone vaginal opening.


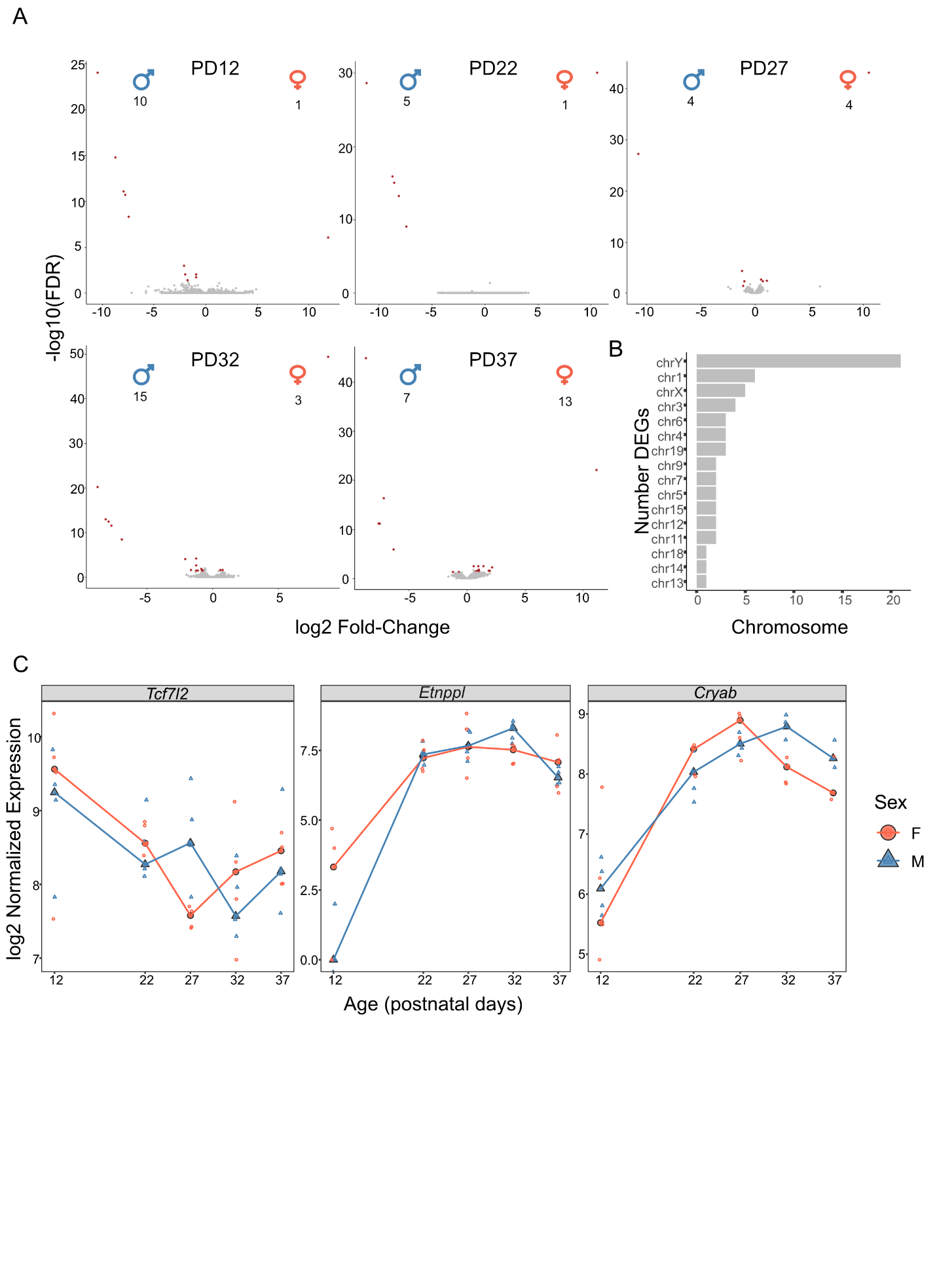


**Supplementary Figure S5. Overview of sex differences across each pubertal timepoint. A) Volcano plots of sex differences at each time point.** The X-axis is the fold-change of each gene, and the Y-axis is the -log10(FDR-adjusted p-value). Genes with positive fold-changes are female-biased, and genes with negative fold-changes are male-biased. B) Barplot of the chromosomal distribution of sex differences at any timepoint. C) Gene expression distribution of three sex-biased genes that were previously associated with puberty.  The X-axis is age, and the Y-axis is log2-transformed RUVseq and ERCC-spike in normalized counts. Red lines and circles represent female samples, while blue lines and triangles represent male samples.


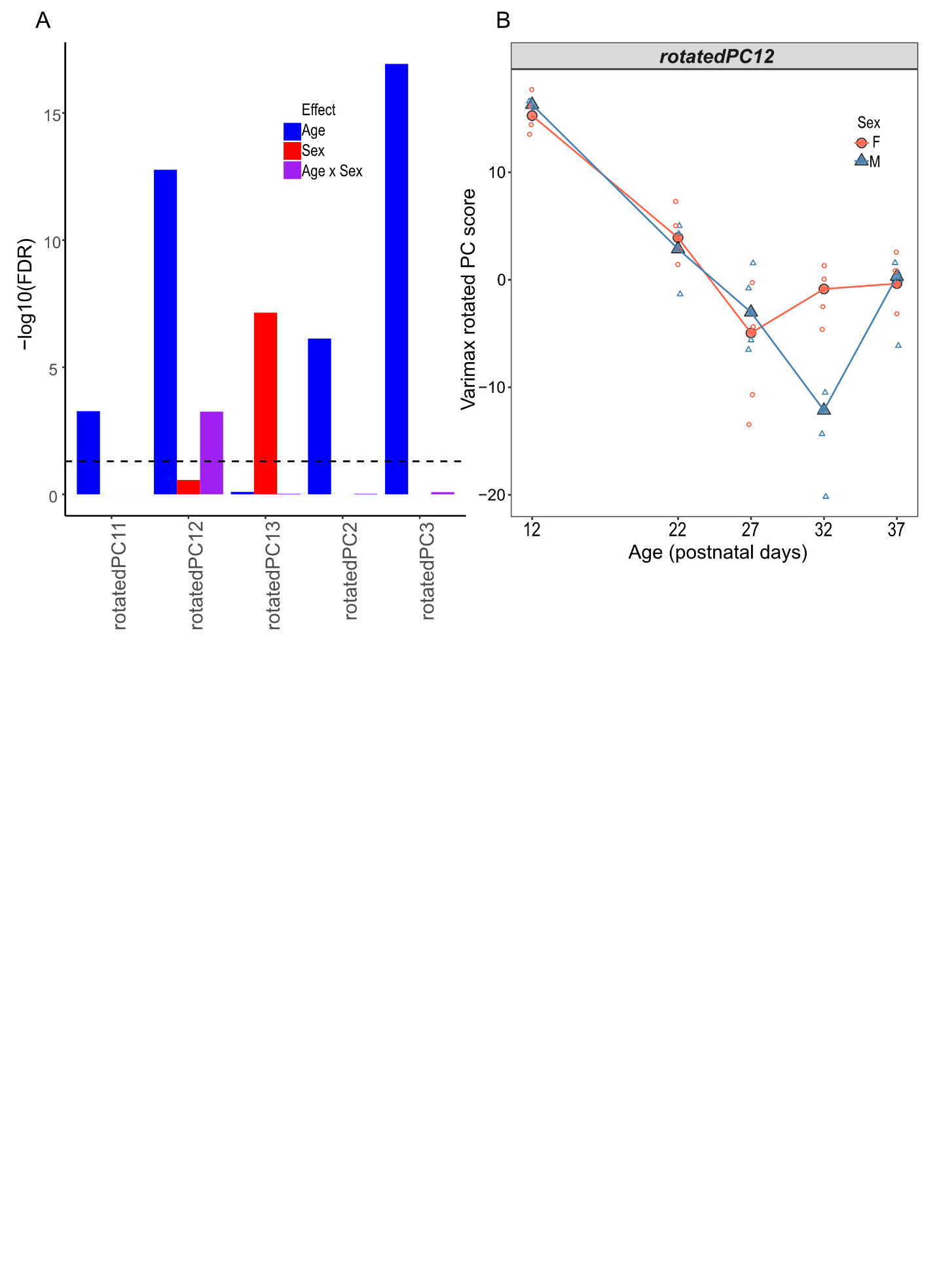


**Supplementary Figure S6.** Identification of an age-by-sex varimax rotated principal component with variable PD12 samples removed. A) Barplot showing the association between each significant varimax rotated PC (vrPC), age, sex, and their interaction. The X-axis shows vrPCs whose scores are associated with age, sex, or an age-by-sex interaction (5/45 total vrPCs). Red bars show the significance of sex, blue bars show the significance of age, and purple bars show the significance of an age-by-sex interaction. B) Distribution of vrPC scores (y-axis) for the rotated principal component with an age-by-sex interaction across each time point (x-axis). Blue points represent male samples and red points represent female samples.


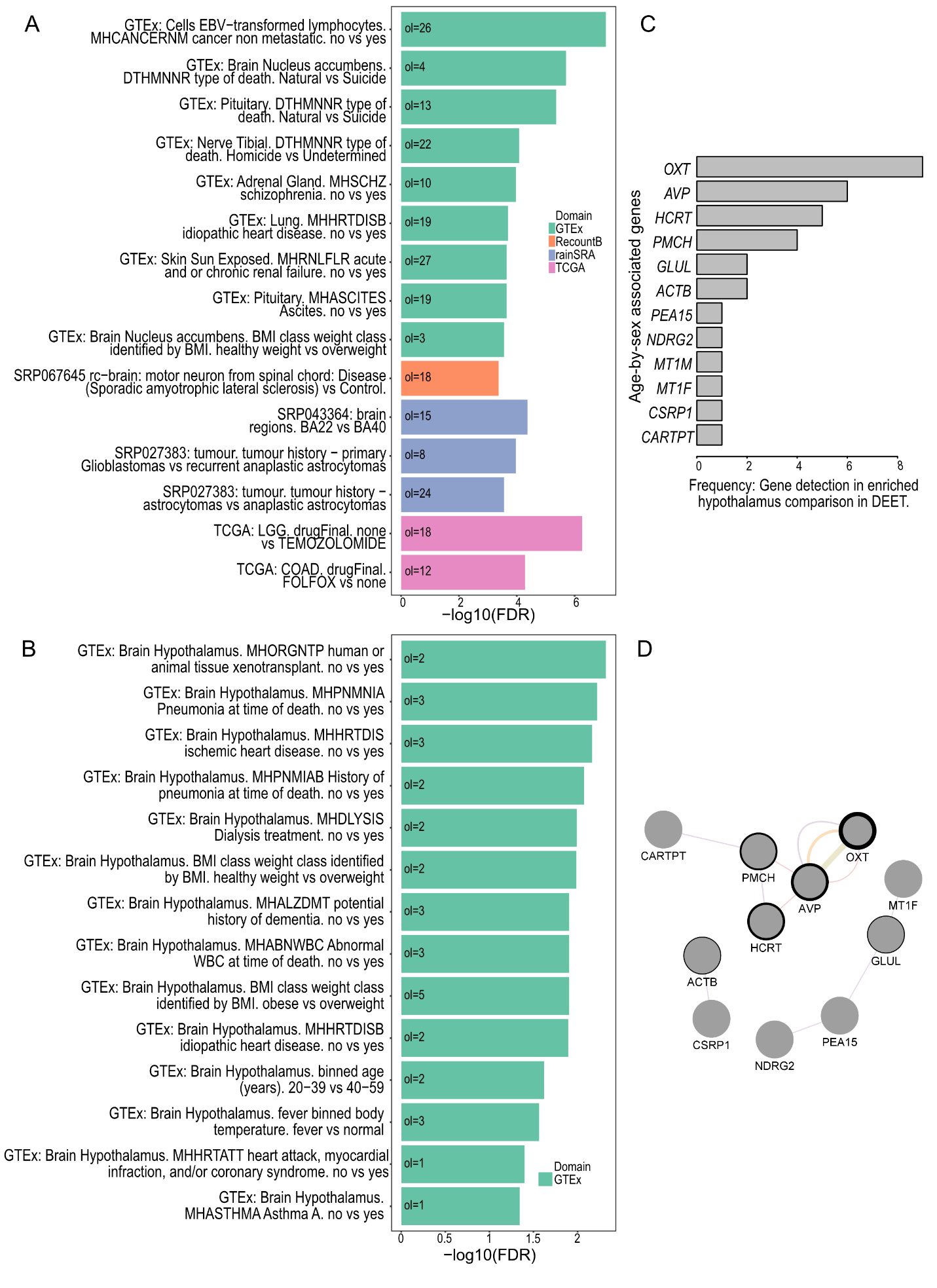


**Supplementary Figure S7. Enrichment of age-by-sex associated genes against 3162 human RNA-seq comparisons stored in the DEET dataset.** Barplot of the top 15 most associated DE comparisons identified using the “DEET_enrich” tool. B) Barplot to the top (up-to) 15 most associated DE comparisons occurring within “Hypothalamus” tissue in DEET. Rows are different comparisons, and the X-axis represents the degree of enrichment for each comparison. Bar colours represent the dataset class where the comparison was sourced from (i.e., recount-brain, TCGA, GTEx, SRA). C) Barplot displaying the frequency of hypothalamus comparisons that age-by-sex associated genes were detected as DE within. D) GeneMANIA of the genes in C). Nodes are genes, and edge-width is the number of hypothalamus comparisons where the gene is DE. Edges represent connections between genes stored within the GeneMANIA dataset. Purple edges represent co-expression, red edges represent protein-protein interactions, orange edges represent predicted interactions, and yellow-green edges represent shared protein domains.


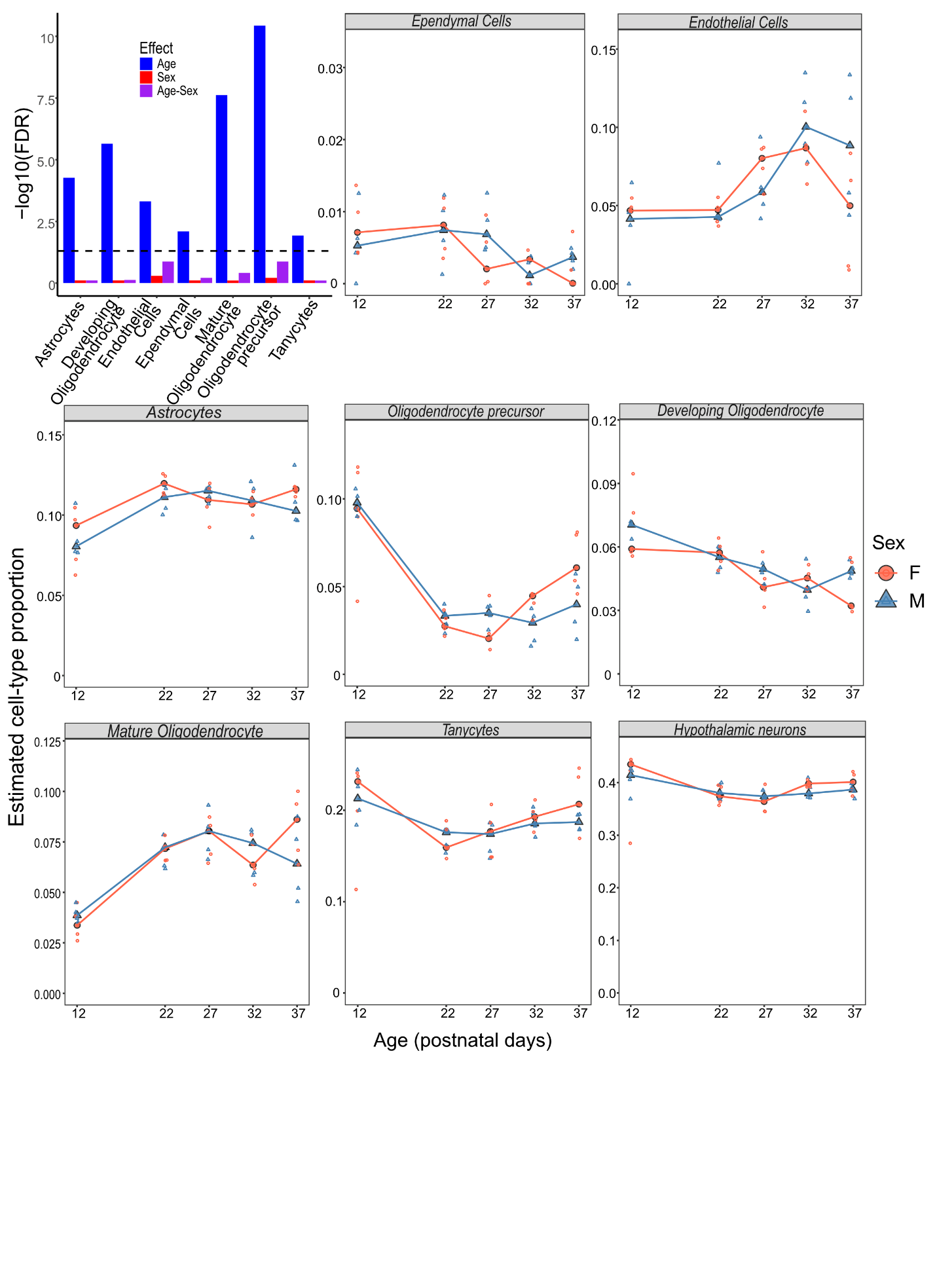


**Supplementary Figure S8. Distribution of cell-type proportions measured from MuSiC-NNLS in cell-types that were predicted to have >3% of the total sample.** The top-leftmost plot displays cell-type proportion association between age, sex, and an age-by-sex interaction across puberty. The X-axis is different cell-types, and Y-axis is the -log10(FDR-adjusted p-value) of a two-way ANOVA. The remaining plots are the cell-type distributions of each cell-type across ages and sex. The X-axis is age, and the Y-axis is the estimated cell-type proportion. Red lines and circles represent female samples, while blue lines and triangles represent male samples.

**
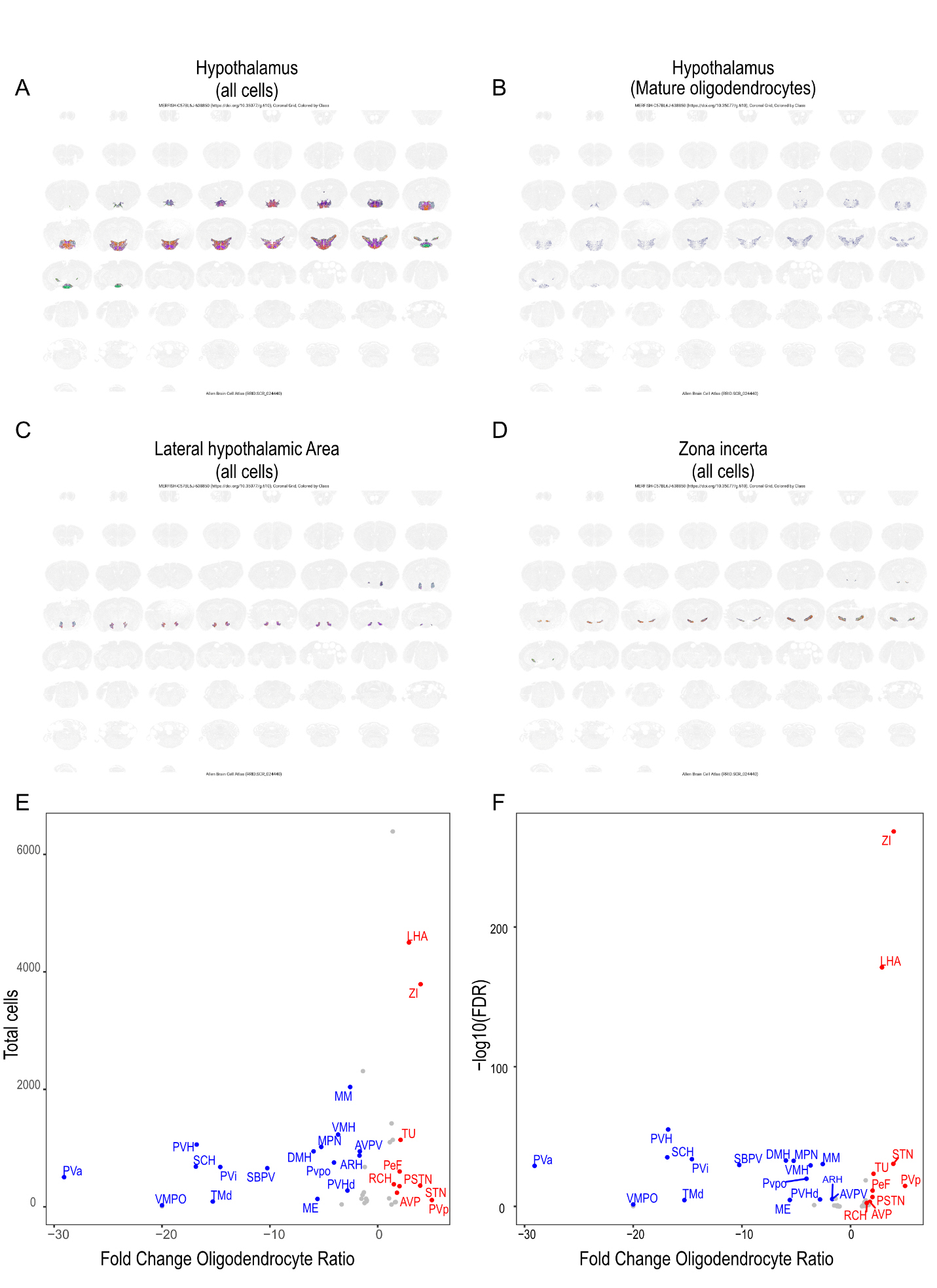
**

**Supplementary Figure 9.** **Distribution of mature oligodendrocytes across the adult mouse hypothalamus using the Allen Brain Cell Atlas (ABCA).** A) Screenshot of the ABCA MERFISH-C57BL6J-638850 dataset, highlighting all cells in the hypothalamus (A), mature oligodendrocytes in the hypothalamus (B), all cells in the lateral hypothalamic area (C), and all cells in the zona incerta (D). E) Volcano plot displaying relationship between the number of mature oligodendrocytes (y-axis) in each region of the hypothalamus and the fold-change of oligodendrocytes (x-axis) in each region of the hypothalamus compared to all the others. F) Volcano plot displayed the -log10(FDR) (y-axis) and fold-change (x-axis) of oligodendrocyte enrichment for each region of the hypothalamus compared to all the others. E) and F), coloured points are regions of the hypothalamus with a fold-change greater than two and a false-discovery rate adjusted p-value of a Fisher’s exact-test < 0.05. Acronyms for hypothalamic regions are designated from the ABCA.


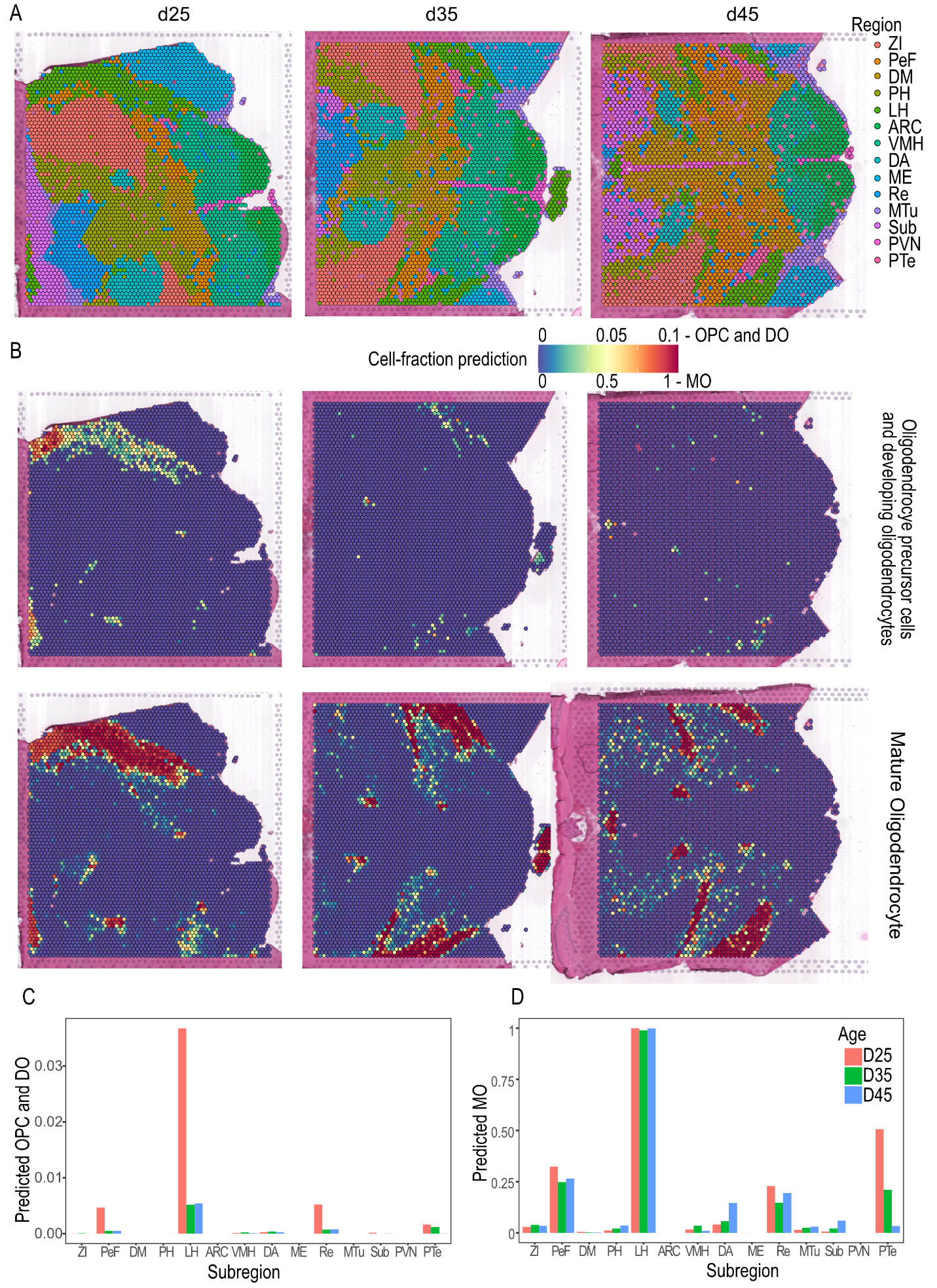


**Supplementary Figure S10.** **Spatially-resolved dynamics of oligodendrocyte precursor cells and mature oligodendrocytes across puberty in the female rat preoptic area.** A) Spatial feature plot of the rat pre-optic area at three post-natal day timepoints. Regions of the pre-optic area were taken from the labels annotated in the original study, Zhou et al., 2022. VHM: ventromedial hypothalamus; PeF: perifornical nucleus; ZI: zona incerta; DM: dorsomedial hypothalamic area; PH: posterior hypothalamic area; LH: lateral hypothalamus; DA: dorsal hypothalamus area; Me: medial amygdaloid nucleus; ARC: arcuate nucleus; MTu: medial tuberal nucleus; Sub: submedius thalamic neuclus; Re: reuniens thalamic nucleus; PVN: paraventricular nucleus; PTe; paraterete nucleus. B) The same spatial feature plots, but spots are now coloured by predicted oligodendrocyte precursor cell and developing oligodendrocytes (OPC and DO) (top) or mature oligodendrocytes (bottom). C and D) Average predicted OPC and DO (C) or MO (D) score in each annotated region of the female rat preoptic area at PD25 (red), PD35 (green), and PD45 (blue).


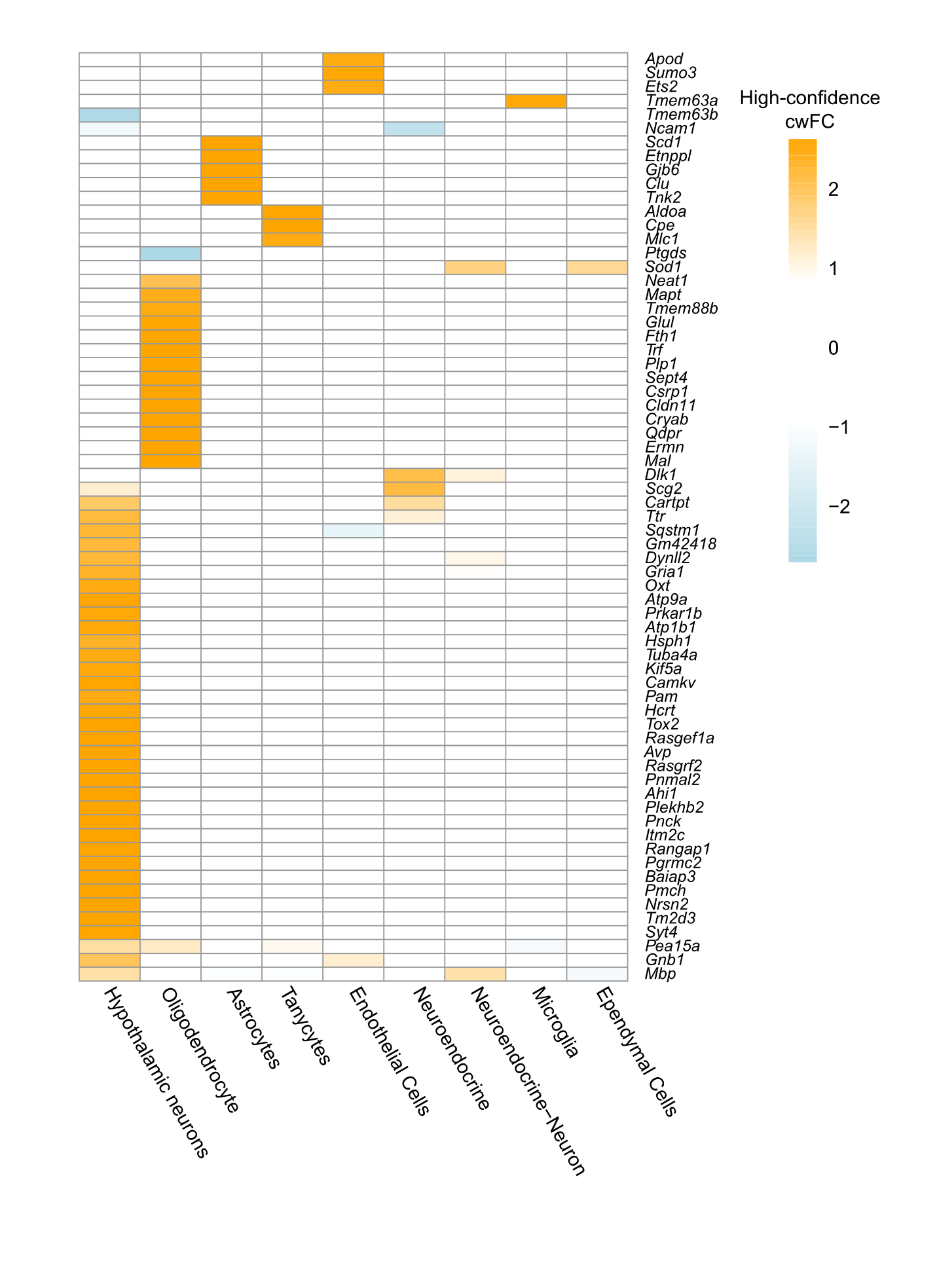


**Supplementary Figure S11. Heatmap of gene-normalized cell-weighted fold-changes (cwFold-changes) of the 129 age-by-sex associated genes and are DE in the complementary direction in the scRNA-seq data.** Rows are genes, and columns are cell-types. Cells in orange represent genes that are more likely to be found in the corresponding cell-type, while cells in blue represent genes that are unlikely to be found in the corresponding cell-type.


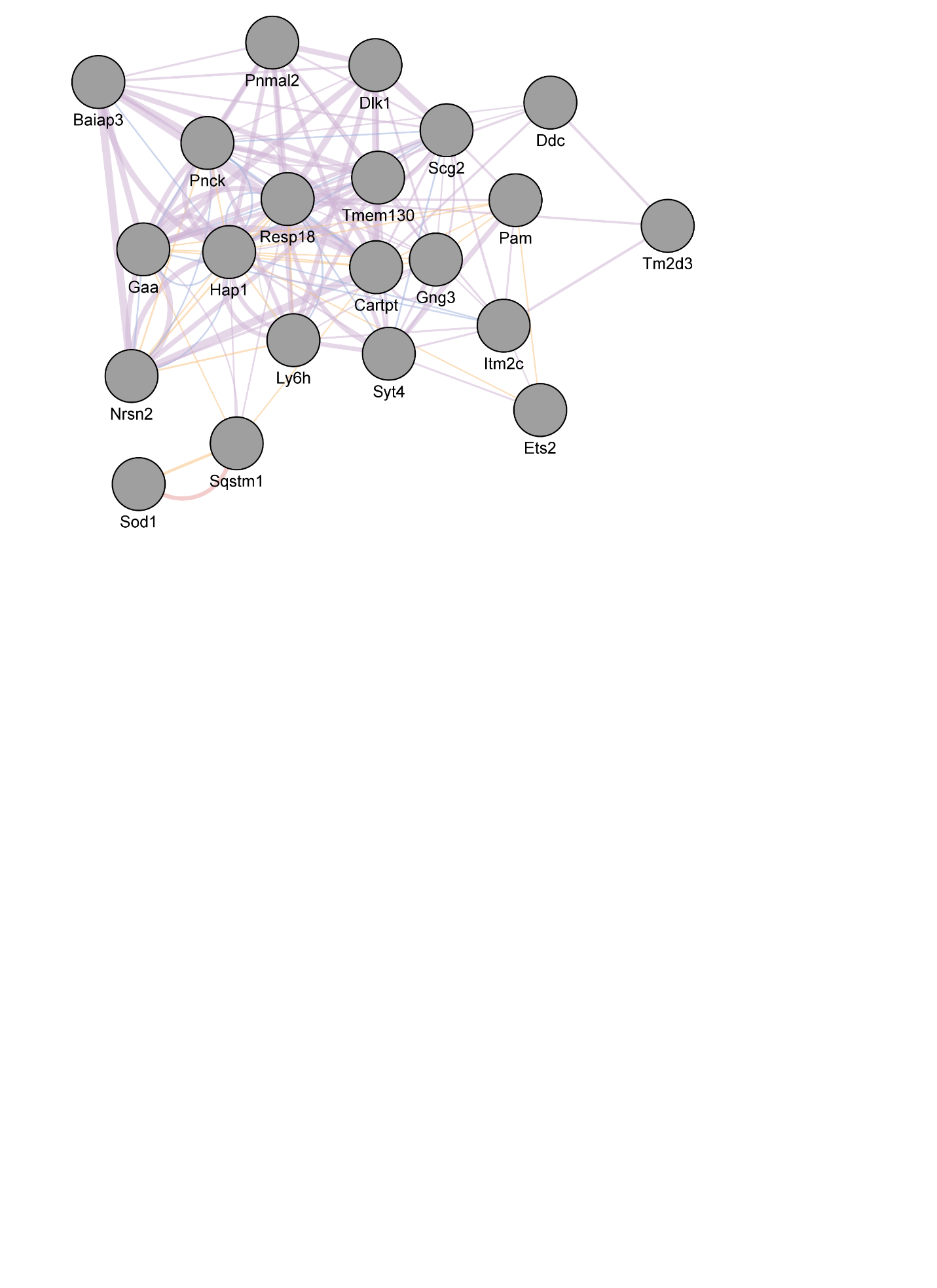


**Supplementary Figure S12. GeneMANIA plot of the 21 neuron- neuroendocrine-mapping age-by-sex associated genes that are detected as translated in hypothalamic LepRb+ neurons in Trap-seq from Allison et al., 2015.** Nodes are genes, and edges are connections between genes pre-computed within the GeneMANIA database. Purple edges are co-expression, blue edges are co-localization, orange edges are predicted interactions, and red edges are validated physical interactions.


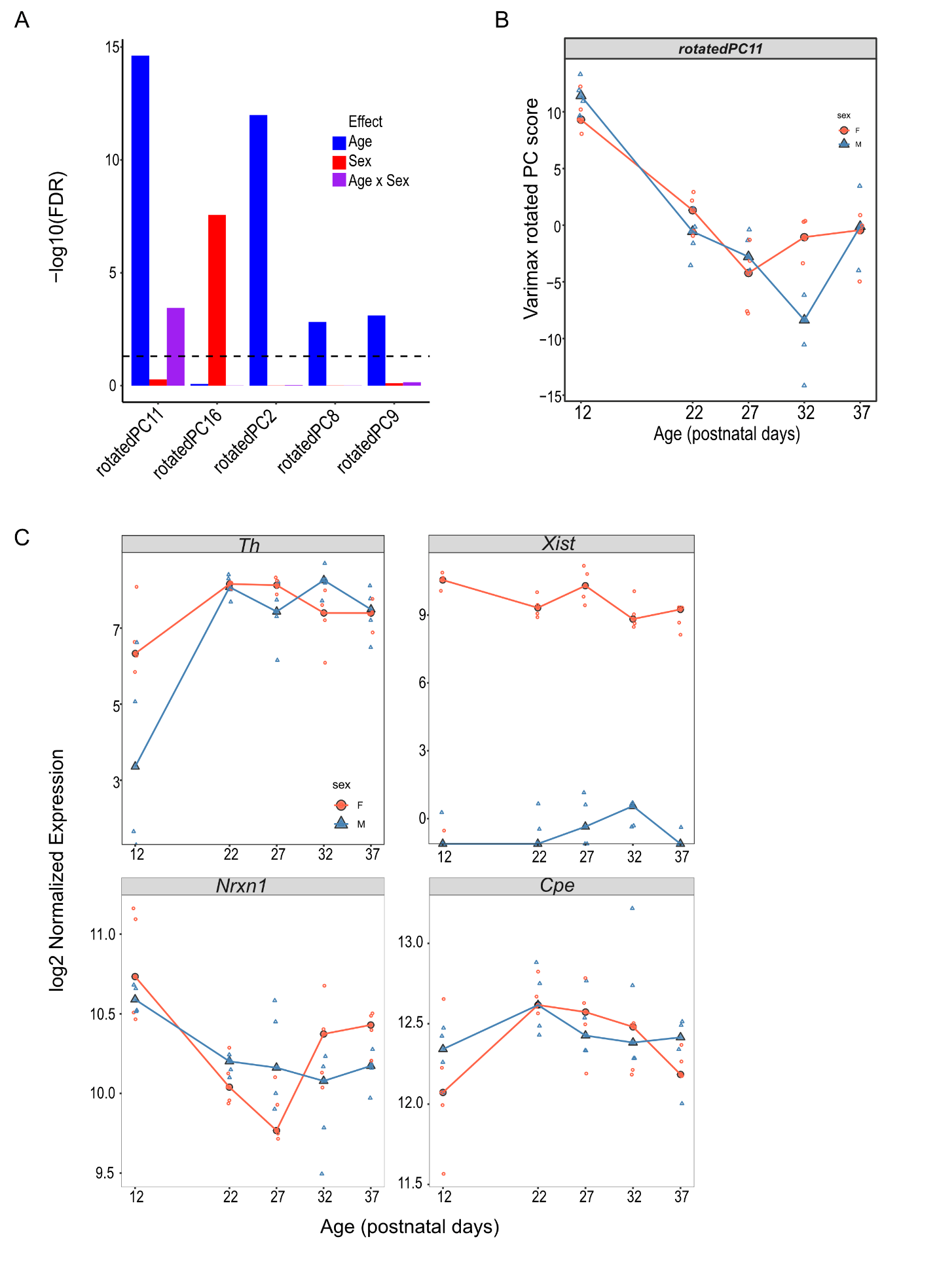


**Supplementary Figure S13.** **Evaluation of an age-by-sex enriched varimax rotated principal component in neuron-adjusted gene expression data.** A) Barplot showing the association between each significant varimax rotated PC (vrPC), age, sex, and their interaction using neuron-adjusted gene expression data. The X-axis shows vrPCs whose scores are associated with age, sex, or an age-by-sex interaction (5/48 total vrPCs). Red bars show the significance of sex, blue bars show the significance of age, and purple bars show the significance of an age-by-sex interaction. B) Distribution of vrPC scores (y-axis) for the rotated principal component with an age-by-sex interaction across each time point (x-axis). C) Distribution of neuron-adjusted normalized gene expression (y-axis) across each time point (x-axis) for four age-by-sex associated genes that have previously been linked to central precocious puberty or hypogonadotropic hypogonadism. In B) and C), blue points represent male samples and red points represent female samples.


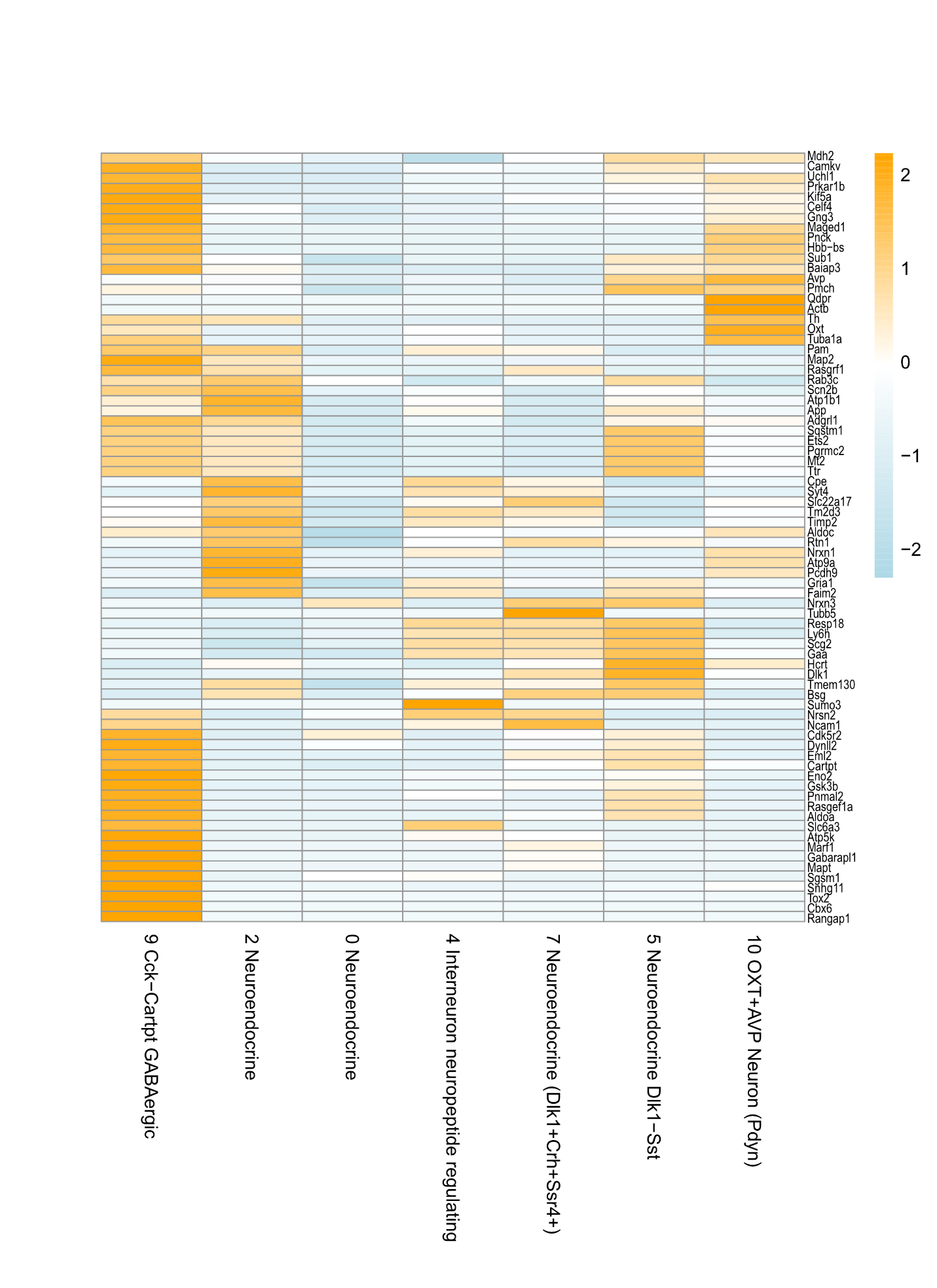


**Supplementary Figure 14. Heatmap of gene-normalized cell-weighted fold-change (Fold-changes) of the 77 age-by-sex associated genes from neuron-adjusted RNA-seq data.** Rows are genes, and columns are cell-types. Cells in orange represent genes that are more likely to be found in the corresponding cell-type, while cells in blue represent genes that are unlikely to be found in the corresponding cell-type.


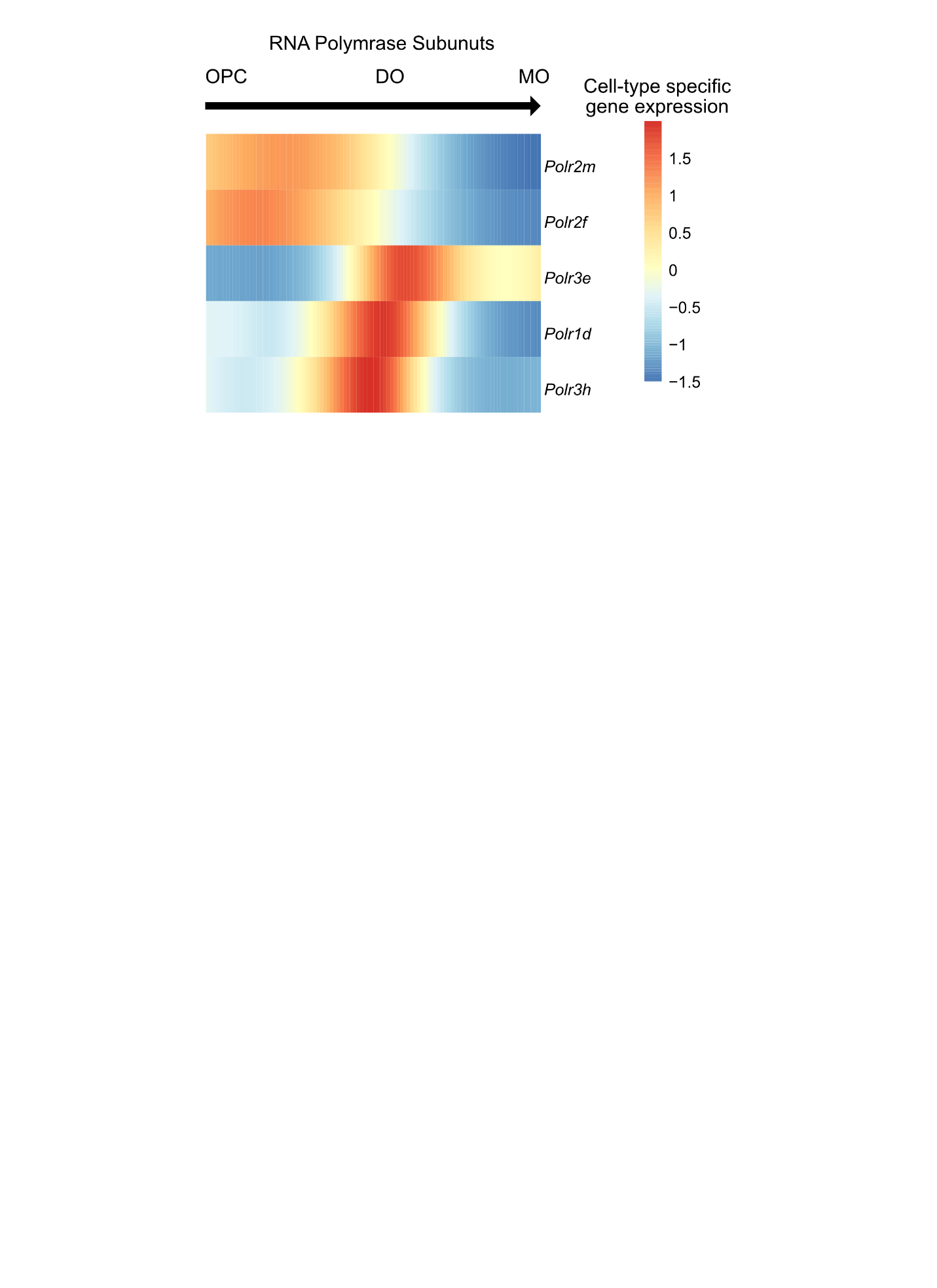


**Supplementary Figure S15. Pseudotime of hypothalamic oligodendrocyte development. Heatmap of RNA polymerase subunit genes associated with pseudotime.** For a gene to be included, it must be associated with an age-by-sex interaction (i.e., varimax 16), mapping to oligodendrocyte precursor cells, developing oligodendrocytes, or mature oligodendrocytes with scMappR, and associate with pseudotime. For B-D, rows are genes associated with pseudotime. Columns are portions of the pseudotime trajectory blocked into 200 smoothers using tradeSeq. Heat is measured by scaling the predicted smoothers with the scale function in R.

**Supplementary Tables**

**Supplementary Table S1**. Summary of RNA-seq sample quality and read mapping using Qualimap.

**Supplementary Table S2**. Correlation and number of cell-types detected (>1% of the total population) between cell-type proportions from RNA-seq deconvolution and cell-type proportions from scRNA-seq data in the mouse hypothalamus.

| Deconvolution Approach | PD12 vs PD14 (scRNA-seq): R^2^ | PD37 vs PD45 (scRNA-seq): R^2^ | All samples vs. PD14 and PD45: R^2^ | Detected cell-types (>1% of population) |
| --- | --- | --- | --- | --- |
| MuSiC | 0.445 | 0.202 | 0.271 | 8 |
| NNLS | 0.743 | 0.638 | 0.696 | 7 |
| CPM | -0.334 | -0.396 | -0.416 | 11 |
| Cibersort | -0.374 | -0.359 | -0.413 | 6 |
| Cibersortx | -0.356 | -0.381 | -0.414 | 6 |
| WGCNA | 0.194 | 0.328 | 0.308 | 5 |
| DCQ | 0.310 | 0.504 | 0.486 | 4 |
| DeconRNAseq | 0.499 | 0.420 | 0.438 | 11 |
| BayesPrism | -0.084 | 0.162 | 0.187 | 6 |
